# Associations between diet quality, epigenetic aging and epigenome in two population-based cohorts

**DOI:** 10.1038/s41467-026-77064-4

**Published:** 2026-08-29

**Authors:** Juliana F. Tavares, Dan Liu, Valentina Talevi, Fabian Eichelmann, Franziska Jannasch, Matthias B. Schulze, N. Ahmad Aziz, Ute Nöthlings, Monique M. B. Breteler

**Affiliations:** 1 Population Health Sciences, German Centre for Neurodegenerative Diseases (DZNE), Bonn, Germany; 2 Department of Molecular Epidemiology, German Institute of Human Nutrition Potsdam-Rehbruecke, Nuthetal, Germany; 3 German Centre for Diabetes Research (DZD), Munich-Neuherberg, Germany; 4 Institute of Nutritional Science, University of Potsdam, Nuthetal, Germany; 5 Department of Neurology, Faculty of Medicine, University of Bonn, Bonn, Germany; 6 University of Bonn, University Hospital Bonn, Institute for Medical Biometry, Informatics and Epidemiology (IMBIE), Bonn, Germany; 7 Institute of Nutritional and Food Sciences – Nutritional Epidemiology, University of Bonn, Bonn, Germany

**Keywords:** Epidemiology, Nutrition, DNA methylation

## Abstract

Several dietary patterns have been associated with better health outcomes. However, the extent to which adherence to different so-called healthy diets overlaps, whether these dietary patterns are equally beneficial, and whether they are associated with similar DNA methylation profiles, remains unclear. Therefore, we investigate the overlap in adherence to ten diet quality scores, and examine the associations of these scores with both biological aging markers and DNA methylation profiles. We use data from the Rhineland Study, a large population-based cohort, and validated our findings using corresponding data from the EPIC-Potsdam cohort. Here, we show minimal overlap of participants classified in the highest quartile (top 25%) of adherence across different diet quality scores. Adherence to a healthy dietary pattern is associated with reduced epigenetic aging, although associations differed in magnitude across diet quality scores. Different dietary patterns are associated with distinct methylation profiles, which however largely converged onto the same biological pathways. Our findings suggest that general adherence to a healthy dietary pattern could promote health through similar epigenetic mechanisms, despite variations in dietary composition.

## Abstract (web summary)

The specific healthy diet may matter less than eating healthily overall. In two German cohorts, several healthy diets were each linked to slower biological aging, with distinct DNA methylation patterns that converged on shared pathways.

## Introduction

With a staggering 11 million global deaths linked to unhealthy eating habits per year—outpacing the toll of smoking and other modifiable risk factors—improving diet quality stands as a cost-effective approach for promoting healthy aging and preventing chronic diseases^1,2^. However, much of the existing research in the field of nutrition tends to concentrate on individual foods and nutrients, lacking a comprehensive perspective on the overall health implications of dietary choices. A focus on dietary patterns, which encompass a wide range of nutrients, foods, and beverages, can offer a more cohesive perspective on how diet influences health outcomes^3^. Commonly used methods to measure adherence to dietary patterns involve hypothesis-driven approaches, including diet quality scores, which measure the extent to which an individual’s diet aligns with current nutritional knowledge or guidelines for disease prevention^4,5^. Several diet quality scores have been developed—for example, the Alternate Healthy Eating Index (AHEI)^6^, Mediterranean-style diet score (MDS)^7^, Dietary Approaches to Stop Hypertension (DASH)^8^, Mediterranean–DASH Intervention for Neurodegenerative Delay (MIND) diet^9^, Nordic diet score^10^, and the Dietary Inflammatory Index (DII)^11^. These diet quality scores have been previously associated with a variety of chronic diseases and mortality^12–14^. Additionally, plant-based diets as assessed by Plant-based Diet Indices (i.e., overall PDI, healthful PDI, and unhealthful PDI), and the EAT–Lancet diet, are receiving increasing attention because of their benefits to both human health and environmental sustainability^15–21^. Recent large-scale epidemiological studies investigating the association between eight dietary patterns and major chronic diseases as well as healthy aging parameters, found that adherence to a healthy diet was associated with a decreased risk of major chronic diseases and greater odds of healthy aging, although their relationship with specific health outcomes varied^22,23^. Because these diet quality scores partially capture shared dietary behaviors, individuals may score highly on more than one pattern at the same time. Although each score emphasizes different dietary components, they share common features, such as higher intake of fruits, vegetables, and whole grains, thus resulting in overlapping representations of overall diet quality. However, the extent to which adherence to various healthy dietary patterns overlaps, and whether they affect health outcomes through similar or distinct molecular mechanisms, has not been explicitly examined.

The molecular mechanisms through which diet quality affects various health outcomes remain largely unknown, yet DNA methylation changes have been suggested to be involved^24,25^. Epigenetic modifications to DNA represent a molecular mechanism through which environmental exposures, including diet, may interact with the genome^26^. Assessing the relationship between diet quality and the epigenome may thus reveal pathways underlying the biological mechanisms between diet quality and health outcomes. Indeed, recent work suggests that diet quality is associated with subtle differences in DNA methylation across the epigenome^27,28^, which in part can be captured by epigenetic clocks that estimate biological age based on DNA methylation patterns^29–34^. Previous investigations have primarily linked individual food items and nutrients (i.e., red meat, fruits and vegetables, plasma carotenoids) with epigenetic clocks and the epigenome, yielding inconsistent results^32,33,35,36^. More recent studies have found that higher adherence to healthy dietary patterns correlated with slower biological aging^37,38^. In addition, a meta-analysis of epigenome-wide association studies (EWAS) of five US and European population-based cohorts using the Illumina 450 K methylation array, which measures 450,000 CpG sites, showed that adherence to MDS and AHEI correlated with differential DNA methylation patterns across the epigenome^27^, and that these diet-associated DNA methylation changes were associated with a lower risk of all-cause mortality^27^. However, whether different dietary patterns (i.e., MDS, DASH, MIND, AHEI, Nordic, EAT–Lancet, PDI, hPDI, uPDI, and DII) have distinct effects on biological aging and DNA methylation has not yet been established. Moreover, few EWAS of dietary patterns have been performed using the Human Infinium MethylationEPIC array that encompasses an extensive coverage of ~850,000 CpG sites across the genome. Importantly, whether diet-induced DNA methylation changes converge on distinct biological pathways that eventually contribute to various health outcomes remains unclear.

We investigated several commonly used, hypothesis-driven diet quality scores with regard to their impact on the methylome and epigenetic markers of biological aging across the adult lifespan. We included both diet quality scores exclusively defined by their assumed beneficial effects on human health, and more recently proposed dietary patterns that aim to address human health as well as environmental sustainability (EAT–Lancet). Specifically, leveraging data from a large population-based cohort study in Germany, the Rhineland Study, we (1) examined the relationship of ten hypothesis-driven diet quality scores with second- and third-generation epigenetic indicators of biological age, (2) performed EWAS studies on all these diet quality scores to identify differential DNA methylation profiles across the entire methylome, and (3) conducted cross-omics functional analyses of diet-associated DNA methylation sites to reveal biological insights into the mechanisms underlying the effects of diet quality on health. Importantly, we replicated our analyses using corresponding data from the population-based EPIC-Potsdam cohort.

## Results

### Sample characteristics

**Fig. 1:**
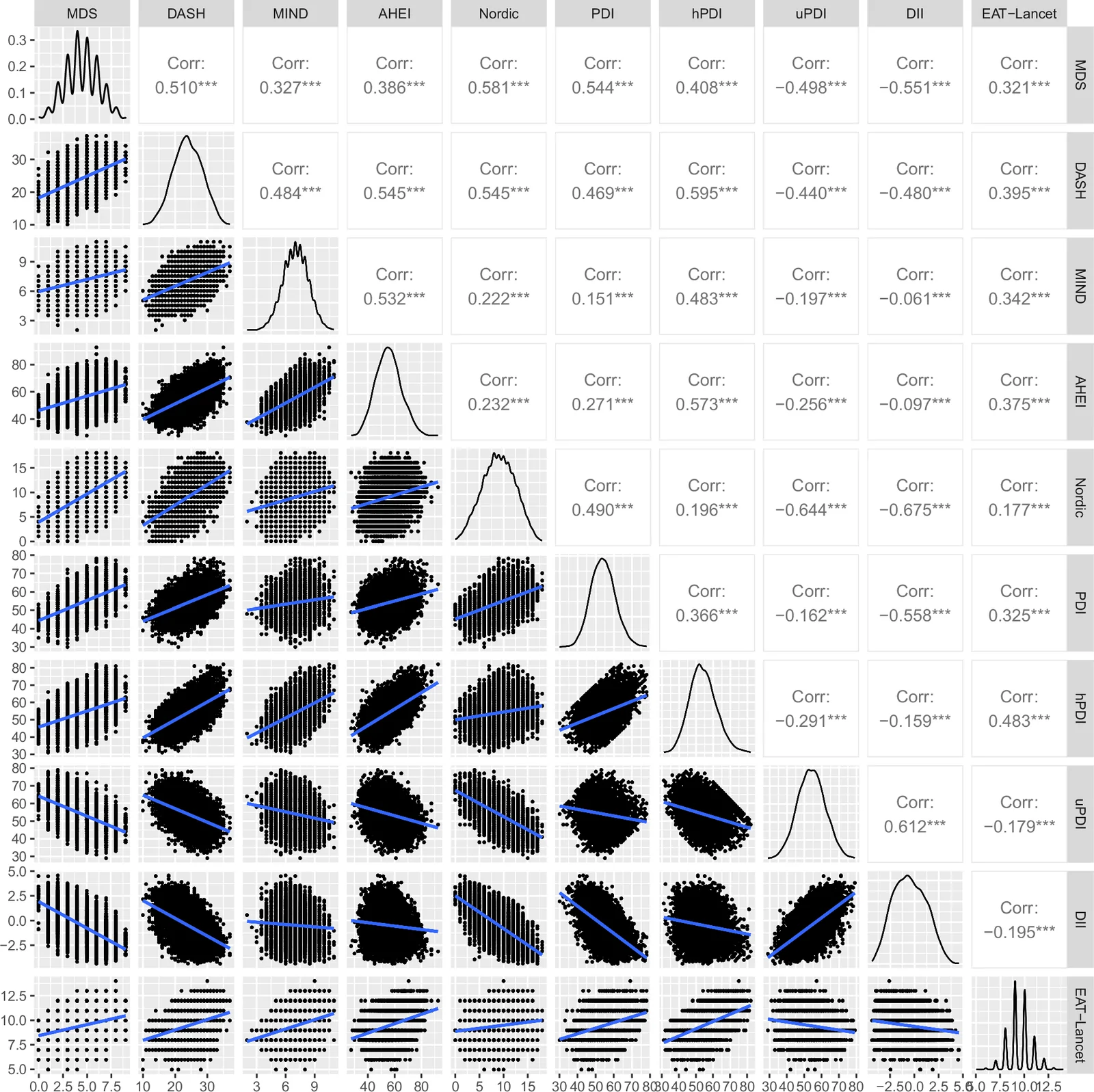
Correlation across diet-quality scores in the Rhineland Study. Correlation matrix with scatter plots and density distributions of diet-quality scores adherence (*n* = 6470 participants). For all scores except uPDI and DII, higher values indicate greater adherence to a healthier diet; for uPDI and DII, higher values indicate a less healthy or more pro-inflammatory diet. The correlation matrix is shown in the upper right triangle, with Pearson’s correlation coefficient (*r*) describing the relationship between each pair of scores. Correlations were tested using two-sided Pearson correlation tests; significance codes (*P < 0.05, **P < 0.01, ***P < 0.001). Scatter plots are shown in the lower left triangle, with fitted regression lines, and histograms of each diet-quality score are shown on the diagonal. Exact correlation coefficients and *P* values are provided in Supplementary Data 2. MDS Mediterranean Diet Score, DASH Dietary Approaches to Stop Hypertension, MIND Mediterranean–DASH Intervention for Neurodegenerative Delay, AHEI Alternate Healthy Eating Index, PDI Plant-based Diet Index, hPDI Healthful Plant-based Diet Index, uPDI Unhealthful Plant-based Diet Index, DII Dietary Inflammatory Index.

**Fig. 2:**
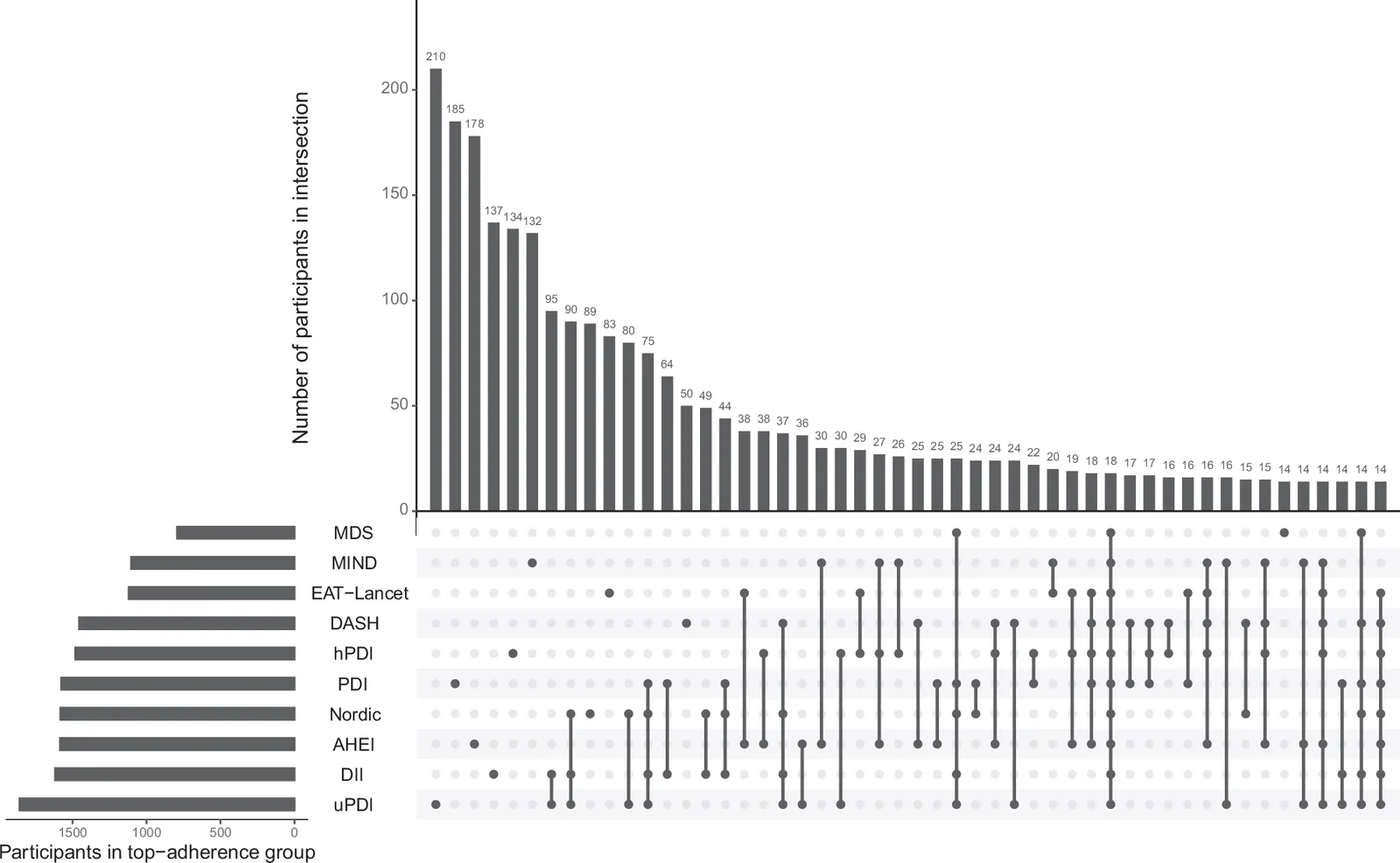
Overlap in high adherence (top 25%) across diet-quality scores in the Rhineland Study. UpSet plot summarizing the overlap in highest adherence (top 25%) across all ten recommendation-based diet-quality scores (*n* = 6470 participants). The plot has three components. The horizontal bar graph at the bottom left (“Participants in top-adherence group”) shows, for each diet-quality score, the total number of participants in the highest quartile of adherence (for the healthy diets) or the lowest quartile of adherence (for the unhealthy diets, uPDI and DII). The dot matrix at the bottom right indicates which combination of diet-quality scores defines each intersection: a single filled dot denotes participants in the top-adherence group for that score alone, whereas multiple filled dots connected by a vertical line denote participants who are simultaneously in the top-adherence group for all the connected scores. The vertical bar graph at the top (“Number of participants in intersection”) shows the number of participants in each corresponding intersection, aligned above the matrix column that defines it. Intersections are ordered from most to least frequent. MDS Mediterranean Diet Score, DASH Dietary Approaches to Stop Hypertension, MIND Mediterranean–DASH Intervention for Neurodegenerative Delay, AHEI Alternate Healthy Eating Index, PDI Plant-based Diet Index, hPDI Healthful Plant-based Diet Index, uPDI Unhealthful Plant-based Diet Index, DII Dietary Inflammatory Index.

The characteristics of the Rhineland Study sample are presented in Table 1 and Supplementary Data 1. Of the 6470 participants in our analytic sample, 3665 (57%) were women. Participants’ mean (± standard deviation (SD)) age was 56.3 ± 13.6 years (age range: 30–95). In general, all diet quality scores were correlated with each other (Fig. 1 and Supplementary Data 2): the uPDI and DII showed inverse correlations with all other indices but a positive association with each other (*r *= 0.61). Among all healthy dietary patterns, the Nordic diet and MDS scores had the strongest correlation (*r* = 0.58), while the Nordic diet and EAT–Lancet scores had the weakest correlation (*r* = 0.18). The epigenetic aging outcomes (GrimAA, PhenoAA, and DunedinPACE) were positively, albeit weakly, correlated with one another (*r* = 0.22–0.28; Supplementary Fig. 2 and Supplementary Data 3). Only a small number of individuals consistently ranked within the top 25% for diet quality across all measured scores (*n* = 18), or at least nine out of these scores (*n* = 77; Fig. 2), with 1212 participants in the top 25% adherence for at least one of the diet quality scores.

**Table 1:** Overview of the population characteristics^a^

Characteristic		Rhineland study (*n* = 6470)
Age, mean y ± SD		56.3 ± 13.6
Women, *n* (%)		3665 (57)
Education ISCED11, *n* (%)	High	3400 (53)
	Medium	2899 (45)
	Low	112 (2)
BMI, mean kg/m^2^ ± SD		25.9 ± 4.5
BMI, categories, *n* (%)	Normal weight	2964 (46)
	Underweight	73 (1)
	Overweight	3398 (53)
Smoking status, *n* (%)	Never	3050 (47)
	Former	2653 (41)
	Current	767 (12)
Hypertension, *n* (%)		2391 (37)
Diabetes, *n* (%)		336 (5)
Total calories, mean kcals ± SD		2489.2 ± 840.1
Alcohol intake, mean g/day ± SD		19.5 ± 27.6
MDS, mean score ± SD		4.5 ± 1.7
DASH diet, mean score ± SD		24.0 ± 4.4
MIND diet, mean score ± SD		7.0 ± 1.3
AHEI-2010, mean score ± SD		55.5 ± 9.3
Nordic diet, mean score ± SD		9.0 ± 3.4
PDI, mean score ± SD		54.0 ± 6.7
hPDI, mean score ± SD		54.0 ± 7.7
uPDI, mean score ± SD		54.0 ± 7.8
DII, mean score ± SD		−0.5 ± 1.7
EAT–Lancet, mean score ± SD		9.5 ± 1.2
GrimAA, y ± SD		−0.1 ± 7.3
PhenoAA, y ± SD		−0.1 ± 6.4
DunedinPACE, units ± SD		1.0 ± 0.1

*SD* standard deviation, *MDS* Mediterranean Diet Score, *DASH* Dietary Approaches to Stop Hypertension, *MIND* Mediterranean–DASH Intervention for Neurodegenerative Delay, *AHEI* Alternate Healthy Eating Index score, *PDI* Plant-based Diet Index, *hPDI* Healthful Plant-based Diet Index, *uPDI* Unhealthful Plant-based Diet Index, *DII* Dietary Inflammatory Index, *BMI* body mass index, *GrimAA* DNA methylation GrimAge Acceleration, *PhenoAA* DNA methylation PhenoAge Acceleration.

^a^Data were expressed as means ± SDs or numbers (percentages).

BMI categories: underweight <18.5 kg/m^2^; normal weight 18.5–24.9 kg/m^2^; overweight ≥25 kg/m^2^. Thirty-five participants were missing information on body mass index.

Education level (self-reported highest educational attainment) was coded as low (lower secondary education or below), middle (upper secondary education to undergraduate university level), and high (postgraduate university study), according to the International Standard Classification of Education (ISCED 2011) and used as a proxy for socioeconomic status.

Recommendation-based diet quality scores range: MDS 0–9; DASH 8–40; MIND 0–14; AHEI-2010 0–100; Nordic 0–18; PDI, hPDI, and uPDI 18–90; EAT–Lancet 0–14.

GrimAA and PhenoAA represent age acceleration, calculated as the residuals from regressing epigenetic age (GrimAge/PhenoAge) on chronological age.

DunedinPACE is a metric of the pace of aging, which quantifies how slowly or rapidly each participant has been aging.

### Association between diet quality and epigenetic aging

**Fig. 3:**
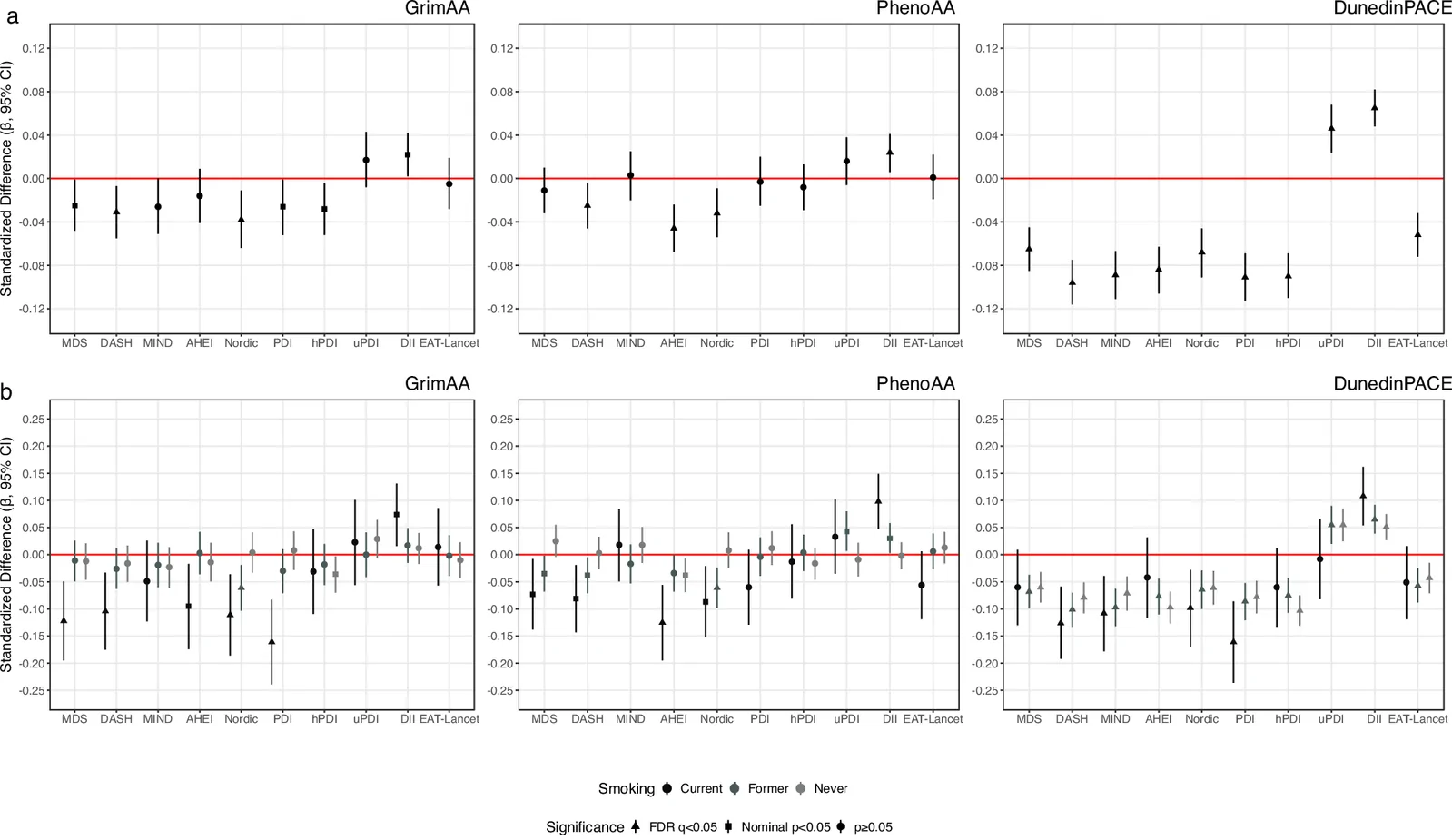
Associations between recommendation-based diet quality scores and epigenetic aging outcomes in the Rhineland Study, overall and stratified by smoking status. The upper forest plots **a** display the β-coefficients and 95% CIs from adjusted multivariable linear regression models, which represent the adjusted mean difference in standardized epigenetic aging outcomes (SD units; z-scores) per 1-SD increase in diet quality (*n* = 6470 participants). Models were adjusted for batch effect, blood cell proportion, sex, smoking status, and total energy intake. Models with DunedinPACE as the outcome were additionally adjusted for chronological age (years). Models testing associations with the DASH, Nordic, EAT–Lancet, PDI, hPDI, and uPDI scores were additionally adjusted for alcohol intake (g/day). The lower forest plots **b** display the β-coefficients and 95% CIs from adjusted multivariable linear regression models, which represent the adjusted mean difference standardized in epigenetic aging outcomes (SD units; z-scores) per 1-SD increase in diet quality among current smokers (black lines; *n* = 767 participants), former smokers (dark gray lines; *n* = 2653 participants), and never smokers (gray lines; *n* = 3050 participants). Models were adjusted for batch effect, blood cell proportion, sex, and total energy intake. Models with DunedinPACE as the outcome were additionally adjusted for chronological age (years). Models testing associations with the DASH, Nordic, EAT–Lancet, PDI, hPDI, and uPDI scores were additionally adjusted for alcohol intake (g/day). *P* values were derived from two-sided tests of the regression coefficients, with no assumption of one-sided effects. To account for multiple comparisons, false discovery rate (FDR) correction was applied using the Benjamini–Hochberg procedure within each epigenetic aging outcome across diet quality scores. Point shapes indicate a graded level of statistical evidence after multiple-testing adjustment, distinguishing robust from suggestive associations: triangles denote BH–FDR *q* < 0.05, squares denote nominal *P *< 0.05 but *q* ≥ 0.05, and circles denote *P* ≥ 0.05. Exact *P*- and *q* values for all associations are provided in Supplementary Data 2 and 8. GrimAA DNA methylation GrimAge Acceleration, PhenoAA DNA methylation PhenoAge Acceleration, DunedinPACE DNA methylation Pace of Aging, MDS Mediterranean Diet Score, DASH Dietary Approaches to Stop Hypertension, MIND Mediterranean–DASH Intervention for Neurodegenerative Delay, AHEI Alternate Healthy Eating Index, PDI Plant-based Diet Index, hPDI Healthful Plant-based Diet Index, uPDI Unhealthful Plant-based Diet Index, DII Dietary Inflammatory Index, FDR false discovery rate.

In the Rhineland Study, higher adherence to the healthy dietary patterns, including MDS, DASH, MIND, AHEI-2010, Nordic diet, PDI, and hPDI, tended to be associated with lower GrimAA, PhenoAA, and DunedinPACE, while higher adherence to unhealthy dietary patterns (i.e., uPDI and DII) tended to be associated with higher values across these outcomes (Fig. 3a and Supplementary Data 4). Notably, all ten diet quality scores were significantly associated with DunedinPACE after Benjamini–Hochberg false discovery rate (FDR) correction. After FDR correction within each epigenetic aging outcome, the DASH and Nordic diet scores were consistently and significantly associated with all three epigenetic aging metrics. Specifically, one SD increase in the DASH score (4.4 points) was associated with 0.03 SD reduction in GrimAA (95% CI: −0.06 to −0.01), 0.03 SD reduction in PhenoAA (95% CI: −0.05 to −0.01) and 0.10 SD reduction in DunedinPACE (95% CI: −0.12 to −0.08). Similarly, a 1-SD increase in the Nordic diet score (3.4 points) was associated with 0.04 SD decrease in GrimAA (95% CI: −0.06 to −0.01), 0.03 SD reduction in PhenoAA (95% CI: −0.05 to −0.01), and 0.07 SD reduction in DunedinPACE (95% CI: −0.09 to −0.05). Conversely, one SD increase in DII (1.7 units) was associated with a 0.02 SD increase in PhenoAA (95% CI: 0.01–0.04), 0.07 SD increase in DunedinPACE (95% CI: 0.05–0.08), and showed a nominally significant positive association with GrimAA (0.02 SD per SD, 95% CI: 0.01–0.04) that did not remain significant after FDR correction. There was no association between EAT–Lancet diet score and GrimAA or PhenoAA epigenetic aging metrics (Fig. 3a and Supplementary Data 4). The analyses of the diet quality scores in quartiles of the distribution aligned with the results of the continuous analyses (Supplementary Data 5). Adjustment for body mass index (BMI) slightly attenuated the associations between the diet quality scores and epigenetic age acceleration metrics (Supplementary Fig. 3 and Supplementary Data 4, Model 2). Similarly, further adjustment for objectively measured physical activity (*n* = 3209 participants with accelerometer data) attenuated these associations, which appears attributable to the reduced sample size rather than confounding, as similar attenuation was observed when restricting the sample without adjustment for physical activity (Supplementary Data 4, Model 3). When excluding participants with prevalent hypertension (Model 4; *n* = 4017) or diabetes (Model 5; *n* = 6034), the associations remained consistent in direction and magnitude with the main findings, suggesting that prevalent cardiometabolic disease does not substantially confound our results (Supplementary Data 4). Stratified analyses showed similar associations between diet quality and epigenetic aging metrics for both sexes (Supplementary Fig. 4 and Supplementary Data 6), and for people with and without obesity (Supplementary Fig. 5 and Supplementary Data 7).

Interestingly, higher adherence to MDS, DASH, AHEI, Nordic, or PDI, and lower adherence to DII, was associated with lower GrimAA, PhenoAA, and DunedinPACE among current smokers, but not in former or never smokers (Fig. 3b and Supplementary Data 8).

### Association between diet quality and epigenome

**Fig. 4:**
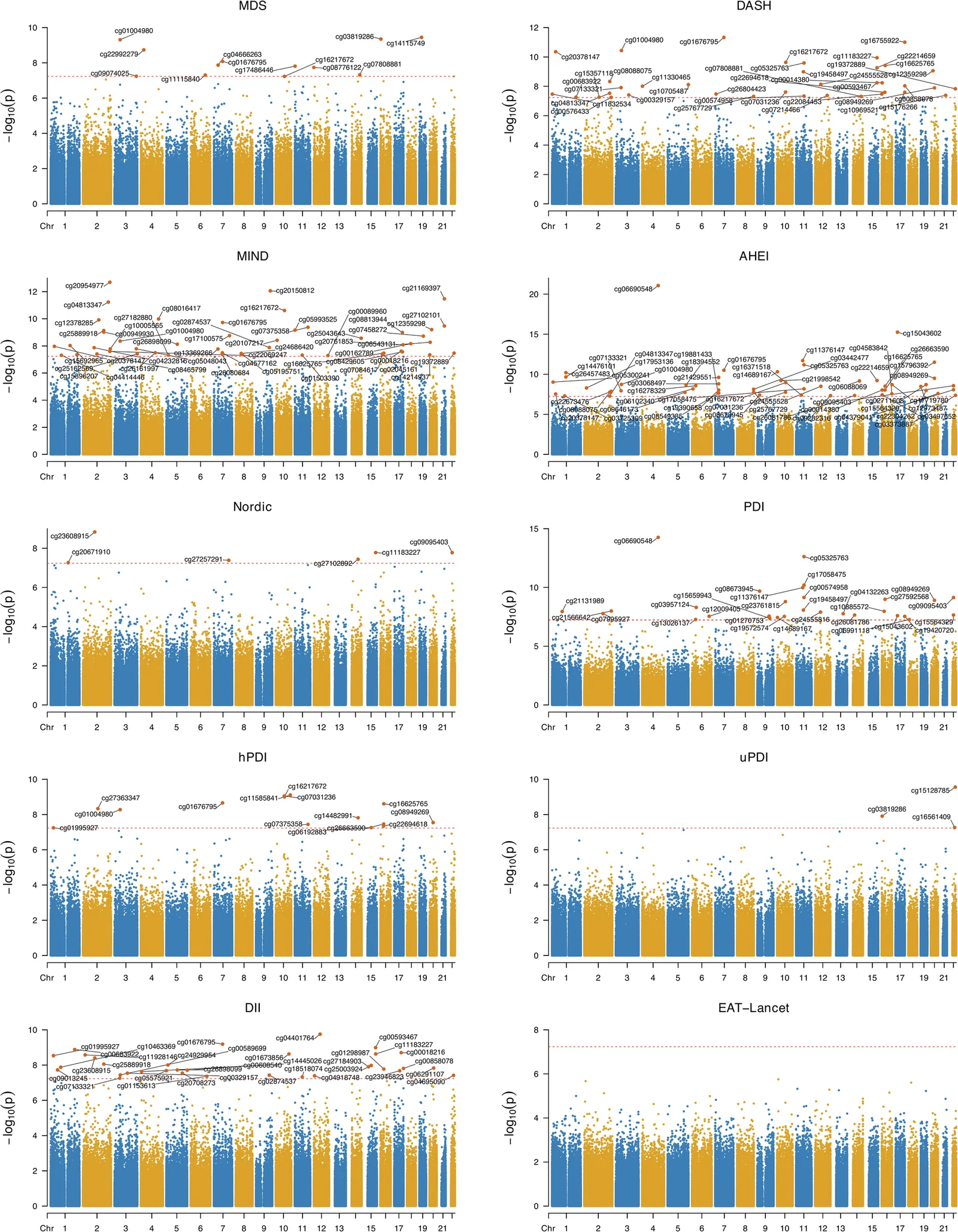
Manhattan plots of the epigenome-wide associations of diet quality scores in the Rhineland Study. Manhattan plots for each recommendation-based diet quality score (*n* = 5768 participants). Results are plotted as negative log-transformed *P* values (*y* axis) across the genome (*x* axis). *P* values were derived from two-sided tests of the regression coefficients in multivariable linear models, adjusted for age, sex, batch effects, blood cell proportion, the first ten genetic principal components to account for population stratification, and smoking status. The red horizontal line represents the epigenome-wide significance based on the Bonferroni threshold (5.95 × 10⁻⁸, 0.05 divided by the number of CpGs tested). The top CpGs of each chromosome were annotated. The full epigenome-wide summary statistics are available via Zenodo (https://doi.org/10.5281/zenodo.20511424). MDS Mediterranean Diet Score, DASH Dietary Approaches to Stop Hypertension, MIND Mediterranean–DASH Intervention for Neurodegenerative Delay, AHEI Alternate Healthy Eating Index, PDI Plant-based Diet Index, hPDI Healthful Plant-based Diet Index, uPDI Unhealthful Plant-based Diet Index, DII Dietary Inflammatory Index, Chr Chromosome.

EWAS analyses revealed that, except for EAT–Lancet, all diet quality scores were associated with DNA methylation levels across the whole genome (Fig. 4). The number of epigenome-wide significant CpG sites ranged from 54 for AHEI-2010 to 3 for uPDI (*P* < 5.95 × 10⁻⁸) (Supplementary Data 9). Of note, half of the identified CpGs were only present on the methylationEPIC v1.0 array, but not on the methylation450K array, and could thus not be identified in earlier studies (Supplementary Data 10). In general, healthy dietary patterns mostly exhibited negative β values. Consistent with this, for uPDI and DII, we observed the opposite trend (Supplementary Fig. 6).

### Validation of findings in an independent cohort

To validate our results, we conducted an independent replication analysis using data from 1034 participants of the EPIC-Potsdam cohort. The mean age of the participants was 49.9 years (SD = 8.9), with 62% being women. The age range was 35–64 years for women and 40–64 years for men. Detailed sample characteristics are presented in Supplementary Data 11.

Overall, the associations between diet quality scores and epigenetic age acceleration metrics (GrimAA and PhenoAA) in the EPIC-Potsdam (Supplementary Fig. 7 and Supplementary Data 12) were consistent with those observed in the Rhineland Study, both in direction and magnitude. This cross-cohort correspondence was further supported by high concordance of effect estimates across diet scores (Supplementary Fig. 8), with Pearson correlations ranging from *r* = 0.79–0.83 for GrimAA and *r* = 0.83–0.91 for PhenoAA across Models. To further quantify the overall associations, we performed an inverse-variance weighted meta-analysis of results from the Rhineland Study and EPIC-Potsdam. The meta-analysis confirmed the patterns observed in each cohort independently, reinforcing the consistency of our findings (Supplementary Fig. 7 and Supplementary Data 13). In particular, the pooled estimates for the DASH, Nordic, AHEI, and DII scores remained statistically significant across both metrics of epigenetic age acceleration after BH–FDR correction. The stratified analyses also mirrored the findings from the Rhineland Study, with no subgroup differences for BMI and sex (Supplementary Data 14 and 15). Among current smokers, higher adherence to several healthy dietary patterns (DASH, MIND, AHEI, PDI) and lower adherence to DII were associated with lower GrimAA after BH–FDR correction (*q* < 0.05), whereas associations with PhenoAA were nominally significant but did not survive BH–FDR correction (Supplementary Fig. 9 and Supplementary Data 16).

Of the 240 epigenome-wide significant CpGs identified in the Rhineland Study across nine dietary patterns, 39 were successfully replicated (*P* value < 0.05 with consistent effect estimate directions, Supplementary Data 17). Among these, the uPDI (67%), AHEI (30%), and DASH (22%) dietary indices showed the highest replication rates (Supplementary Fig. 10). Furthermore, even among CpGs that did not reach nominal significance (*P* > 0.05) in the replication cohort, more than 50% of the top CpGs retained consistent effect directions with the discovery cohort (Supplementary Figs. 10 and 11 and Supplementary Data 17). Beta-beta correlation analyses of the epigenome-wide significant CpGs (identified in the discovery cohort) further supported the consistency of the findings between the Rhineland Study and EPIC-Potsdam (*r* = 0.54, *P* < 0.001) (Supplementary Fig. 12). Stratified analyses by diet quality score revealed varying degrees of correlation, with the highest reproducibility observed for CpGs associated with AHEI (*r *= 0.85), uPDI (*r* = 0.92), and DII (*r* = 0.67), while lower correlations were seen for CpGs linked to Nordic (*r* = 0.09) and MIND (*r* = 0.25) diets (Supplementary Fig. 13).

No single CpG reached the epigenome-wide significance threshold (*P* < 5.95 × 10⁻⁸) for the EAT–Lancet diet score in either the discovery (Rhineland Study) or the replication (EPIC-Potsdam) cohort. Although we found CpGs associated with EAT–Lancet adherence in both cohorts at the suggestive significance level (*P* < 1 × 10⁻⁵), these completely differed between the two cohorts (Supplementary Fig. 14), without a significant correlation between the beta values across CpGs (*r* = 0.007).

### Cross-omics functional analyses of diet-associated DNA methylation

**Fig. 5:**
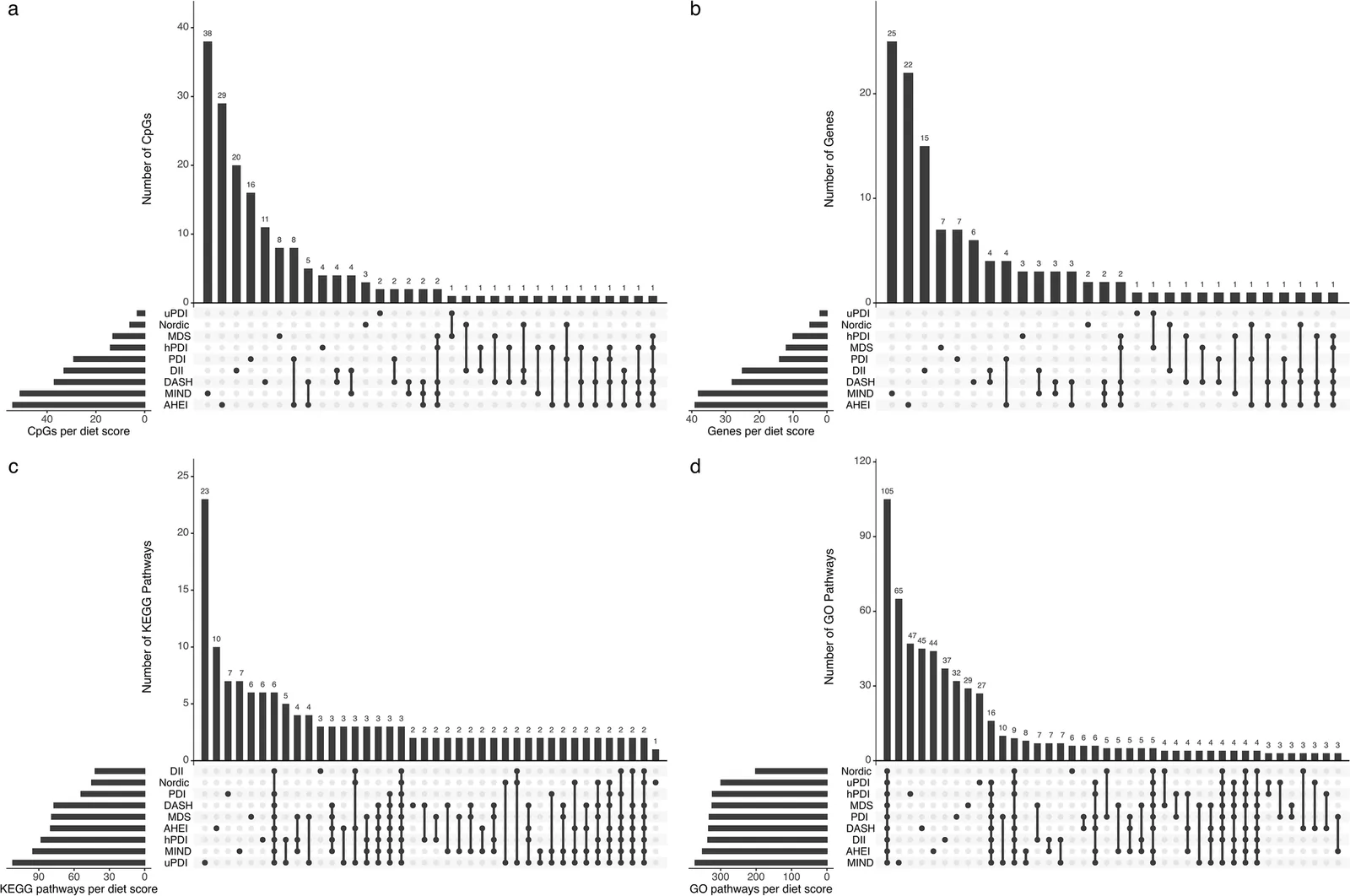
Overlap in diet-associated DNA methylation (epigenome-wide significantly associated CpGs), their mapped genes, and related KEGG and GO pathways, across different recommendation-based diet quality scores. The upper UpSet panels summarize the differentially methylated CpGs (**a**) and mapped genes (**b**) that overlap across the recommendation-based diet-quality scores. The bottom left horizontal bar graph represents the total number of CpGs or mapped genes for each diet quality score. The dots in each panel’s matrix represent unique and overlapping differentially methylated CpGs or their mapped genes: a single dot denotes items unique to one score, while connected dots designate CpGs (or mapped genes) shared across the connected diet quality scores. The top bar graph in each panel recapitulates the number of differentially methylated CpGs or mapped genes for each unique or overlapping combination. The underlying overlap data are provided in Supplementary Data 18 (CpGs) and 19 (mapped genes). The lower UpSet panels summarize the number of KEGG (**c**) and GO (**d**) pathways that overlap across the recommendation-based diet-quality scores. The bottom left horizontal bar graph represents the total number of KEGG or GO pathways for each diet quality score. The dots in each panel’s matrix represent unique and overlapping KEGG or GO pathways: a single dot denotes pathways unique to one score, while connected dots designate pathways shared across the connected diet quality scores. The top bar graph in each panel recapitulates the number of KEGG or GO pathways for each unique recommendation-based diet-quality score or overlapping combinations. The underlying overlap data are provided in Supplementary Data 20 (KEGG) and 21 (GO). MDS Mediterranean Diet Score, DASH Dietary Approaches to Stop Hypertension, MIND Mediterranean–DASH Intervention for Neurodegenerative Delay, AHEI Alternate Healthy Eating Index, PDI Plant-based Diet Index, hPDI Healthful Plant-based Diet Index, uPDI Unhealthful Plant-based Diet Index, DII Dietary Inflammatory Index, KEGG Kyoto Encyclopedia of Genes and Genomes, GO Gene Ontology.

In the Rhineland Study discovery cohort, we observed minimal overlap in the associated CpGs (Fig. 5a and Supplementary Data 18) and mapped genes (Fig. 5b and Supplementary Data 19) across the 9 diet quality scores. In contrast, these distinct CpGs/mapped genes were involved in biological pathways that overlapped for more than 70% (Fig. 5c, d and Supplementary Fig. 15). These included the Kyoto Encyclopedia of Genes and Genomes (KEGG) pathways adherens junction, axon guidance, calcium signaling pathway, endocytosis, and the MAPK signaling pathway (Supplementary Data 20 and Supplementary Fig. 16), as well as the gene ontology (GO) pathways actin binding, cell morphogenesis, and neurogenesis (Supplementary Data 21 and Supplementary Fig. 17). In addition, we identified diet quality scores-specific biological pathways that may underlie the distinct health effects of the corresponding diet. For instance, aortic valve development and morphogenesis, heart valve development and morphogenesis were uniquely associated with the DASH diet (Supplementary Data 21); whereas cognition, learning, or memory, and central nervous system neuron axonogenesis were uniquely associated with the MIND diet (Supplementary Data 21). Despite the minimal overlap in the diet-associated CpGs, the beta values of these CpGs were moderately correlated across diet scores (Supplementary Fig. 18).

A lookup in the EWAS Catalog revealed that several of the CpGs associated with MDS, DASH, MIND, AHEI, hPDI, and DII in our data had been previously associated with AHEI in other cohorts (Supplementary Data 22). Specifically, we examined overlap with the two prior EWAS of diet quality that applied similar hypothesis-driven scoring to population-based cohorts^27,28^. Three CpGs in our discovery cohort overlapped with the 24 AHEI-associated CpGs reported by Do et al.^28^: cg01676795 (*POR*), which was replicated in EPIC-Potsdam (consistent effect direction and *P* < 0.05) for DASH, DII, hPDI, and MIND; cg01004980 (*PRKAR2A*), which was partially replicated (consistent effect direction, *P* > 0.05) for AHEI, MDS, and MIND; and cg20954977 (*B3GNT7*), which was partially replicated for MIND. For cg01676795 and cg01004980, the overlap included the same diet score (AHEI), whereas cg20954977 was associated with MIND rather than AHEI in our cohort. No overlap was observed with the 10 MDS- or 24 AHEI-associated CpGs reported by Ma et al.^27^. This may reflect differences in methylation array coverage, as our study used the MethylationEPIC v1 array, whereas the two previous studies used the 450 K array. Moreover, these distinct dietary pattern-related CpGs have been previously linked to a wide range of diet-related traits (i.e., alcohol consumption, smoking), cardiometabolic traits (i.e., type II diabetes, BMI, waist circumference, CRP, insulin and glucose levels, blood pressure), lipid levels (i.e., HDL, LDL, triglycerides), protein levels, as well as mortality (Supplementary Data 22). Interestingly, their mapped genes have been linked to several brain aging-related traits (including brain morphology, cortical surface area, subcortical volume, and general cognitive ability (Supplementary Data 23).

**Fig. 6:**
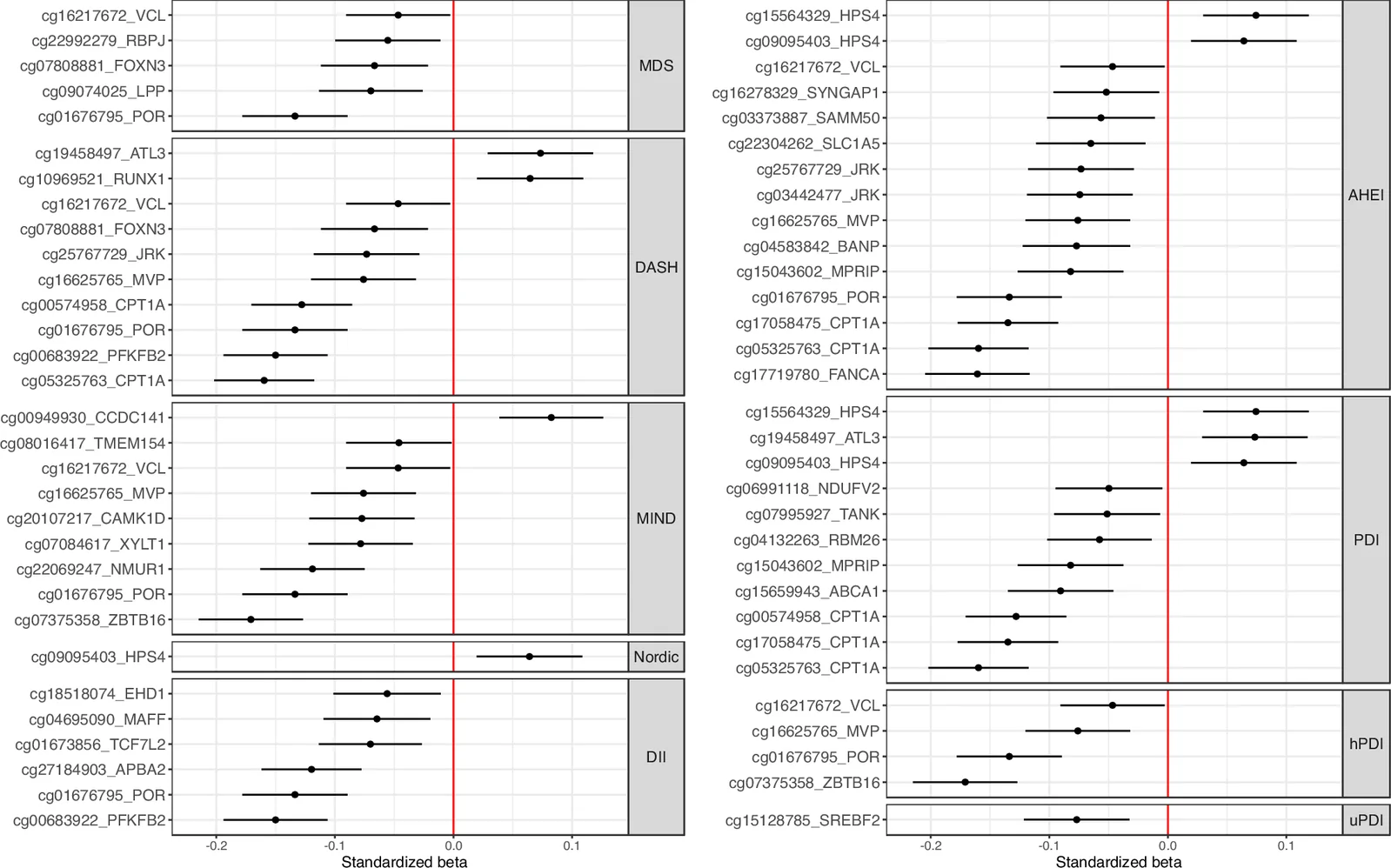
Diet-specific relations between associated CpGs and their mapped gene expression levels. Forest plots showing the effect estimate of diet-associated CpGs that were significantly associated with the expression level of their mapped gene, clustered by diet quality score. Each row represents a CpG and its mapped gene, labeled as probe identifier_gene symbol (e.g., cg01676795_POR). Points represent the standardized beta estimate from the following multivariable linear model: Gene_y_ ~ intercept + CpG_x_ + age + sex + blood cell proportion + 10 PCs + smoking status + batch effects. Error bars represent 95% confidence intervals around the β estimate. *P* values were derived from two-sided tests of the regression coefficients. Analyses were performed in *n* = 1923 participants of the Rhineland Study with both DNA methylation and gene expression data available. The corresponding effect estimates, 95% confidence intervals, and exact *P* values are provided in Supplementary Data 24. *MDS* Mediterranean Diet Score, DASH Dietary Approaches to Stop Hypertension, MIND Mediterranean–DASH Intervention for Neurodegenerative Delay, AHEI Alternate Healthy Eating Index, PDI Plant-based Diet Index, hPDI Healthful Plant-based Diet Index, uPDI Unhealthful Plant-based Diet Index, DII Dietary Inflammatory Index.

Diet-associated CpGs were associated with the expression levels of their mapped genes in both in silico analyses and our expression quantitative trait methylation (eQTM) analysis. For example, cg00574958 and cg17058475 (*CPT1A*), cg20954977 (*B3GNT7*), cg07458272 (*KIAA0355*), and cg02711608 (*SLC1A5*) were previously associated with the expression levels of the mapped genes in the BIOS database (Supplementary Data 10). Using individual-level gene expression data from the Rhineland Study, including expression data on 93 out of 126 mapped genes, we further extended these findings. We found that 33 diet-associated CpGs were associated with their mapped gene expression levels (Fig. 6 and Supplementary Data 24), including genes with important metabolic functions (i.e*., POR, SLC1A5, CPT1A*)^39^. Moreover, we observed that diet quality scores were associated with a small subset of these candidate genes, including *CPT1A, ABCA1*, *ATL3*, and *PFKFB2* (Supplementary Fig. 19). Specifically, three distinct diet quality scores (i.e., DASH, AHEI, and PDI) were related to *CPT1A* gene expression, thereby supporting the significance of this gene in metabolic pathways.

Through the mQTLdb database, we uncovered several genetic variants (i.e., methylation quantitative trait loci (mQTLs), Supplementary Data 8), which exhibited associations with the methylation levels of CpGs linked to the various dietary patterns. However, the majority of diet differential CpGs were not associated with any mQTLs. This is probably because the currently available mQTLs databases are all based on the 450 K array, whereas half of the hits we identified are exclusive to the MethylationEPIC v1.0 array. In addition, we performed enrichment analyses to assess whether EWAS-identified CpGs for each diet quality score were over-represented among CpGs with known mQTLs, restricting both EWAS-identified CpGs and background CpGs to those present on the Illumina 450K array and tested in our EWAS (conducted on the 850 K array). We found that none of the dietary trait-associated CpGs showed significant enrichment for mQTLs after correction for multiple testing (all FDR-adjusted *P* values > 0.05; Supplementary Data 25). These results suggest that the identified CpGs overlapping the 450 K array are not predominantly driven by known genetic regulation. As mQTL resources for the EPIC/850 K array become available in the future, similar analyses could be extended to CpGs unique to the 850 K platform.

## Discussion

Our results demonstrate that stronger adherence to healthier diets, as defined by multiple diet quality scores, was consistently associated with slower epigenetic aging in the general population, as captured by both second-generation clocks (GrimAge and PhenoAge) and the third-generation DunedinPACE metric measuring the pace of biological aging. This trend was more prominent in current smokers. Intriguingly, different dietary patterns were associated with unique DNA methylation patterns, which however largely targeted similar biological pathways. These pathways predominantly involve processes related to cell structure, signaling, and metabolism, which are crucial in aging and chronic disease development. Additionally, we identified diet-specific pathways aligning with reported health claims for certain diets (e.g., DASH, MIND). Furthermore, these diet-associated CpGs have also been linked with aging-related traits, suggesting a functional connection between these epigenetic modifications and health outcomes.

Our observation that adherence to healthier diets was associated with slower biological aging aligns with findings from previous studies. Wang et al. found that adherence to a healthy diet, assessed through eight dietary patterns, is generally linked to a lower risk of major chronic diseases^22^. More recently, Tessier et al. showed that higher adherence to healthy diets was associated with a higher likelihood of healthy aging, as assessed according to measures of cognitive, physical, and mental health, as well as living to 70 years of age free of chronic diseases^23^. Kim et al. demonstrated a link between a higher DASH score and reduced epigenetic age acceleration^37^. Similarly, Kresovich et al. reported an inverse relationship between four diet quality scores, including DASH, AHEI-2010, and a modified MDS, and epigenetic age acceleration in a cohort of non-Hispanic white females^38^. Our study significantly extends those previous findings by not only reaffirming the connection between diet quality and markers of epigenetic aging, which focus on a limited number of CpG sites, but also by showing that the relationship between diet quality and the epigenome extends beyond these markers. Moreover, by demonstrating consistent associations across both second-generation clocks (reflecting accumulated biological age) and DunedinPACE (reflecting the pace of aging), our findings suggest that diet quality influences multiple dimensions of the biological aging process. Replication in the EPIC-Potsdam cohort further supported these findings by confirming a similar pattern of associations between diet quality scores and epigenetic aging indicators, along with partial replication at the epigenome-wide level. The stronger association between diet quality scores and epigenetic aging acceleration in current smokers, which was also replicated in the EPIC-Potsdam, was observed despite current smokers being less likely to fall in the healthiest quartile across most diet scores (Supplementary Data 26), suggesting that this pattern is unlikely to be explained by higher overall diet quality among smokers. Sensitivity analyses excluding participants with prevalent hypertension or diabetes yielded associations consistent in direction and magnitude with the main findings, suggesting that the observed relationships are not driven by confounding due to disease-related dietary changes. The partial attenuation of some associations in these analyses, as well as after adjustment for BMI, is consistent with cardiometabolic factors potentially lying on the causal pathway between diet quality and epigenetic aging rather than acting solely as confounders^34,40,41^.

Our EWAS and cross-omics analyses in the discovery cohort illuminate the molecular mechanisms through which diet influences health outcomes. We found minimal overlap in the specific CpGs or genes associated with different diet quality scores, yet a substantial overlap in the associated biological pathways. Thus, while different healthy diets may influence distinct sets of epigenetic markers, they converge on similar biological processes. These shared pathways, including pathways related to cell signaling, metabolism, and neurogenesis, point to common molecular mechanisms underlying the link between nutrition and health. In silico analyses revealed that many diet-associated CpGs have been previously related to a wide range of health outcomes, supporting their relevance in mediating the health effects of diet. In addition, we identified diet-specific biological pathways that may underlie the specific health effects of the corresponding diet. For instance, the DASH diet has been associated with a lower risk of CVDs, and exhibited methylation changes involved in heart function-related pathways. Similarly, MIND-related methylation changes were implicated in central nervous system axonogenesis and cognition-related pathways. From an applied perspective, the convergence of different dietary patterns on overlapping molecular pathways suggests that adopting a broadly healthy diet is likely beneficial at the population level. The fact that multiple, distinct dietary patterns share common epigenetic effects reinforces the public health message that promoting overall healthy eating patterns can have widespread health benefits. However, our finding that certain diet-specific pathways are also implicated—such as those related to cardiovascular health in the DASH diet or cognitive function in the MIND diet—highlights the potential for personalized dietary guidance. For individuals at higher risk of specific diseases, targeted dietary strategies could be developed based on the epigenetic markers and pathways most relevant to their risk profiles. Furthermore, while our eQTM analyses identified significant associations between 33 diet-associated CpGs and their mapped gene expression levels, the majority of identified CpGs did not show such associations in blood. This does not preclude their biological relevance. DNA methylation in blood may serve as a biomarker of systemic dietary exposures, with functional transcriptional consequences occurring in metabolically relevant tissues such as adipose tissue, liver, or the gastrointestinal tract^42^. Moreover, CpGs located in distal enhancers may regulate genes through long-range chromatin interactions rather than the nearest annotated gene, and gene body methylation may primarily affect alternative splicing rather than total expression levels^43^. DNA methylation also serves functions beyond transcriptional regulation, including maintenance of genome stability and chromatin structure^44^. Finally, the limited availability of expression data for mapped genes (93 of 126) may have reduced our power to detect methylation-expression associations.

The variations in the strength of associations between different diet quality scores with epigenetic markers of biological aging and the methylome can be attributed to the structural differences in the conceptualization and calculation of these scores^6–11,15,18^. While many of these scores harbor similarities, such as emphasizing the consumption of fruits, vegetables, and whole grains while limiting the intake of red and processed meats and sugars, they do diverge regarding specific food items or groups that are included or how they are accounted for (i.e., the same food item may be scored differently across different diets)^6–11,15,18^. For instance, the MIND diet^9^ focuses on specific foods that contain nutrients that may support brain health—such as vitamin E, folate, n-3 fatty acids, carotenoids, and flavonoids—commonly found in berries, leafy greens, and moderate amounts of wine.

Similarly, the DASH diet also promotes plant-based foods but also prioritizes specific nutrient targets, such as sodium reduction, to support cardiovascular health^8^. These differences may explain the limited overlap among participants with the highest adherence to various diet quality scores and correspond to the distinct biological pathways associated with specific dietary patterns that may underlie possible diet-specific health effects, highlighting the potential for personalized dietary guidance. Nevertheless, we found comparable associations across different diet quality scores and across the discovery and replication cohorts, supporting the generalizability of the beneficial effects of adherence to a general healthy diet.

Of note, we found little to no association of adherence to the EAT–Lancet diet with second-generation epigenetic aging metrics (GrimAge and PhenoAge) or genome-wide DNA methylation. However, adherence to the EAT–Lancet diet was significantly associated with DunedinPACE, suggesting that this dietary pattern may influence the pace of biological aging despite not showing associations with markers of accumulated biological age. This discrepancy may reflect differences in what these clocks capture: DunedinPACE was designed to measure the current rate of aging based on longitudinal changes in multiple biomarkers, whereas GrimAge and PhenoAge reflect cumulative biological aging burden^32,33,45^. The EAT–Lancet diet’s recommended ranges emphasize environmental sustainability, and unlike the other diet quality scores, set upper intake limits, even for healthy food groups, and establish a minimum intake of zero grams per day for several nutrient-dense food groups^17,18^. These intake ranges may be sufficient to influence the ongoing pace of aging but may not produce the sustained or cumulative effects needed to detectably alter second-generation epigenetic aging markers or specific CpG methylation patterns. Warranting further research to understand how different aspects of biological aging respond to dietary patterns that aim to balance human and planetary health. Although our EAT–Lancet adherence score was operationalized using the 2019 Commission definition, the 2025 update indicates that the planetary health diet pattern is not substantially different, suggesting our conclusions are unlikely to be materially affected^21^.

While our study provides substantial insights, it also has limitations. Our findings are based on a predominantly German population of European ancestry in both cohorts; generalizability to persons with other ancestries or dietary habits remains to be investigated. Moreover, the FFQ-based dietary assessment is subject to measurement error and may not capture all dietary nuances. For example, they may not precisely capture specific or less frequently consumed foods. Despite these limitations, FFQs generally provide sufficient coverage of habitual dietary intake for assessing dietary patterns. Although multiple potential confounders were adjusted for in the present analysis, residual confounding could not be completely ruled out. In addition, although a similar FFQ and DNA methylation assessment were employed between the discovery and replication cohorts, certain differences, particularly the fact that dietary data in EPIC-Potsdam were collected in the 1990s, possibly reflecting different eating patterns at the time, as well as potential regional variations in eating patterns despite both cohorts being from Germany, may account for some observed variance in effect estimates. Despite these nuances, the overall concordance in both direction and magnitude of associations supports their generalizability beyond the Rhineland Study population. Lastly, our study focused on three second- and third-generation epigenetic aging metrics (GrimAA, PhenoAA, and DunedinPACE); other metrics capturing different aspects of biological aging may provide complementary insights. However, all epigenetic aging metrics are based on specific CpG subsets, and our comprehensive EWAS approach examining ~850,000 CpG sites provides broader coverage of diet-methylation relationships across the epigenome.

In conclusion, our results confirm the importance of promoting healthy dietary patterns as a crucial strategy for mitigating the aging process and its associated health risks. Additionally, our findings indicate that the choice of a specific healthy dietary pattern might not be as critical as the overarching adherence to a generally healthy diet. However, it is noteworthy that specific dietary patterns may offer additional benefits, potentially serving as tailored interventions for specific high-risk populations. Finally, the replication of our results in an independent cohort reinforces their generalizability, underscoring their potential impact on the development and implementation of public health programs aimed at promoting healthy aging.

## Methods

This research complies with all relevant ethical regulations. Approval to conduct the Rhineland Study was granted by the ethics committee of the Medical Faculty of the University of Bonn. The EPIC-Potsdam study protocol was approved by the ethics committee of the Medical Society of the State of Brandenburg, Germany. Both studies were conducted in accordance with the International Council for Harmonization Good Clinical Practice standards and the principles of the Declaration of Helsinki. All participants provided written informed consent prior to enrollment.

### Study population

We used cross-sectional baseline data from the Rhineland Study, an ongoing population-based prospective cohort study. The study recruits individuals aged 30 years or older who live in either of two distinct municipal districts in Bonn, Germany, and who have a sufficient command of the German language to provide informed consent. The Rhineland Study participants are predominantly German of Caucasian descent. A primary objective of the Rhineland Study is to identify determinants and markers of healthy aging through a deep-phenotyping approach. At baseline, participants complete an 8-h in-depth multi-domain phenotypic assessment, and various types of biomaterials (including blood, urine, stool, and hair) are collected. The baseline dietary assessment is completed remotely in a web-based format by most participants, within a defined window following the study center visit, during which buffy coat samples for DNA methylation analysis are drawn.

Between May 2016 and November 26, 2021, 8318 participants enrolled in the Rhineland Study. This study is based on the 6470 participants with complete information on diet, epigenetic data, and covariates of interest. Of these, 5768 participants with available genetic data and of European ancestry were further included in our EWAS; the ancestry restriction was applied to reduce confounding by ancestry-specific differences in DNA methylation patterns. Details on the stepwise exclusion of participants with missing or incomplete dietary assessment, epigenetic data, or covariate information are provided in Supplementary Fig. 1.

### Dietary intake and diet quality scores calculation

Participants’ dietary intake was assessed using a semi-quantitative Food Frequency Questionnaire (FFQ). The FFQ inquired about the consumption frequency of specified portion sizes of food and beverage items over the past year^46^. For analysis inclusion, FFQs needed data on at least 80% of food items, consistent with other cohort studies using a similar instrument^47^. Questionnaires not meeting this completeness threshold were considered incomplete and excluded. Missing data (up to 20%) were imputed with MissForest, as detailed elsewhere^48^. Nutrient intake was estimated as the sum of nutrient content across all food items based on data from the German Food Code and Nutrient Database^49^. The FFQ data served as the basis for estimating adherence to the following diet quality scores: MDS, DASH, MIND, AHEI-2010, Nordic, EAT–Lancet, PDI, hPDI, uPDI, and DII^6–11,15,18^. For further details, see Supplementary Methods.

### DNA methylation quantification

Genomic DNA was extracted from buffy coat fractions of anti-coagulated blood samples. DNA methylation levels were measured by Illumina iScan with Illumina’s Human MethylationEPIC BeadChip (v1.0), which measures ~850,000 CpG sites across the genome. Detailed information is provided in the Supplementary Methods.

### Estimation of epigenetic aging

Both our previous work and studies by others have shown that second- and third-generation epigenetic clocks better capture inter-individual variability in biological aging processes and multi-system dysfunction than first-generation clocks, which primarily reflect chronological age^34,50,51^. Therefore, we focused our analyses on second- and third-generation measures, including PhenoAge, GrimAge, and DunedinPACE. We calculated PhenoAge and GrimAge as epigenetic markers of biological age based on the algorithms developed by Levine et al. and Lu et al., using 513 and 1030 CpG sites, respectively^32,33^. The age acceleration estimators GrimAA and PhenoAA were defined as the residuals (in years) that result from regressing the PhenoAge and GrimAge age estimates on chronological age, such that positive values indicate higher biological age than expected for chronological age. DunedinPACE was calculated using the DunedinPACE R package (version 0.99.0; https://github.com/danbelsky/DunedinPACE)^45^. DunedinPACE is a DNA methylation-based measure of the pace of aging that is scaled to a mean of 1 in the reference population and can be interpreted as the rate of biological aging per chronological year (e.g., values > 1 indicate faster aging and values < 1 indicate slower aging).

### Whole blood RNA isolation and gene expression profiling

PAXgene Blood RNA tubes (PreAnalytix/Qiagen) were used to collect fasting blood samples between 7:00 and 9:45 in the morning from an antecubital or dorsal hand vein. Total RNA was isolated according to the manufacturer’s instructions using PAXgene Blood miRNA Kit and following the semi-automatic purification protocol (PreAnalytix/Qiagen). Gene expression sequencing was performed on the NovaSeq 6000 instrument (Illumina) platform. Detailed information on the sequencing protocol and quantification of gene expression levels is provided in the Supplementary methods.

### Covariates

We used questionnaires and interviews to obtain information on participants’ characteristics, including age, sex, history of medical conditions and medication, education, and smoking status. Smoking status was defined as current smoker, former smoker, or never smoker based on self-report. Participants’ weight and height while in underwear by trained staff. Body mass index (BMI) was calculated as weight divided by height squared (kg/m^2^). Physical activity was recorded with accelerometers and reported as average daily energy expenditure in metabolic equivalents (METs) per hour^52^. Total daily energy intake (kcal/day) and alcohol consumption (grams/day) were derived from the FFQ. Hypertension was defined as a self-reported physician diagnosis of hypertension, an average systolic blood pressure ≥140 mmHg and/or diastolic blood pressure ≥90 mmHg, or regular use (i.e., daily, every other day, or weekly during the past year) of antihypertensive drugs as defined by the Anatomical Therapeutic Chemical (ATC) classification system (codes C02, C03, C07, C08, C09). Diabetes was defined as a self-reported physician diagnosis, glycated hemoglobin ≥6.5%, or regular use of anti-diabetic medication (ATC code A10).

### Statistical analyses

The statistical analyses were conducted using R (version 4.2.1, The R Foundation). For sample demographics, we reported the mean and standard deviation (SD) for continuous variables, and the number and percentage for categorical variables. To compare the sample characteristics of the included and excluded participants, we performed binomial logistic regression adjusted for age and sex. The distributions of diet quality scores and age acceleration estimators were examined using histograms, and correlations among the diets were calculated using Pearson correlation coefficients. The overlap of participants in the top 25% for diet quality (highest quartile of MDS, DASH, MIND, AHEI-2010, Nordic Diet, EAT–Lancet, PDI, and hPDI; and lowest quartile of uPDI and DII) across all diet quality scores was illustrated using an UpSet plot.

### The association between dietary quality indices and epigenetic aging

To investigate the relationship between dietary quality indices (independent variables) and three epigenetic aging outcomes (dependent variables: GrimAA, PhenoAA, DunedinPACE), we used multivariable linear regression models. Models were adjusted for sex, smoking status, total energy intake, batch effect (including chip, row, and first five control probe principal components), and blood cell proportion estimated from the same DNA methylation samples^53^. Models with DunedinPACE as the outcome were additionally adjusted for chronological age (years). In the models examining associations with DASH, Nordic, PDI, hPDI, and uPDI scores, we additionally adjusted for alcohol intake (g/day). To evaluate the effect of adiposity and physical activity, we additionally adjusted for BMI and physical activity measured as metabolic-equivalent hours (MET-hours) in sensitivity analyses in sensitivity analysis. To address potential confounding by prevalent cardiometabolic disease, we repeated the main analyses after excluding participants with hypertension or diabetes. In all analyses, the diet quality scores were transformed to have a mean of 0 and a standard deviation of 1. Epigenetic aging outcomes were also standardized (z-scores); therefore, β estimates represent the adjusted mean difference in the outcome (SD units) per 1-SD increase in diet quality.

To determine whether the associations were primarily driven by individuals with the highest diet quality scores, we categorized the continuous diet quality scores into quartiles of their distributions. We then assessed linear trends by using the median value for each quartile category of the diet quality score as a continuous variable in our statistical model. We also evaluated the effect modification of the diet quality scores by sex, smoking status, and BMI, as these factors have been associated with altered DNA methylation patterns and epigenetic age acceleration in prior studies^54–59^. Specifically, interaction terms were added to the models. In the interaction analysis, smoking status was considered as the three self-reported smoking categories (never, former, and current), and BMI was dichotomized into BMI < 25 and ≥25 kg/m^2^. Whenever an interaction term met the nominal significance threshold (*P* ≤ 0.05), we performed a stratified analysis by the respective effect modifier. The effect estimates are presented with corresponding two-sided 95% confidence intervals. To account for multiple comparisons, we controlled for false discovery rate (FDR) using the Benjamini–Hochberg procedure within each epigenetic aging outcome across diet quality scores. Results were considered statistically significant at FDR q < 0.05; nominal *P* values are reported for completeness. To evaluate whether the model assumptions were met, we visually inspected the distribution of the residuals.

### Epigenome-wide association analysis (EWAS) of diet quality scores

We investigated the relationship between diet quality scores (independent variables) and DNA methylation level (dependent variable) across the genome using multiple linear regression, adjusting for age, sex, batch effects (including chip, row and first five control probe principal components), blood cell proportion, the first ten genetic principal components to account for population stratification, smoking status and total energy intake (Model 1). We further adjusted for BMI in model 2. We applied the Bonferroni correction to account for multiple comparisons. An association was considered epigenome-wide significant if its *P* value was smaller than the Bonferroni threshold (5.95 × 10⁻⁸, 0.05 divided by the number of CpGs).

### Validation in an independent cohort

To externally validate our results, we conducted a replication analysis in an independent cohort, the EPIC-Potsdam study. Like the Rhineland Study, the participants of EPIC-Potsdam were predominantly German of Caucasian ancestry. The replication sample was drawn from a randomly selected subcohort of EPIC-Potsdam (*n* = 1147) assembled as part of a nested case-cohort design^60,61^; the 1034 participants included in our replication analysis were those with available methylation data and complete dietary and covariate information. This subsample is broadly representative of the overall EPIC-Potsdam cohort in terms of key demographic characteristics (Supplementary Data 9). This study employed similar methods for the measurement of dietary intake and DNA methylation levels as those used in the Rhineland Study. In EPIC-Potsdam, dietary intake was assessed using a semi-quantitative FFQ. The ten diet quality scores^6–11,15,18^ (MDS, DASH, MIND, AHEI-2010, Nordic, EAT–Lancet, PDI, hPDI, uPDI, and DII) were calculated following the same methods as those in the Rhineland Study, as described in the Supplementary Methods. In EPIC-Potsdam, both dietary assessment and buffy coat collection were performed at baseline. DNA methylation levels in participants were measured using Illumina’s Human MethylationEPIC BeadChip (v1.0), while PhenoAge and GrimAge were calculated using the same established algorithms. The statistical analyses were applied identically to assess the associations between dietary quality indices and epigenetic age acceleration, as well as to perform an epigenome-wide association analysis (EWAS) of diet quality scores.

We conducted a meta-analysis using inverse-variance weighing to estimate the pooled association between dietary patterns and epigenetic age acceleration across cohorts. For each cohort, we obtained beta estimates and their corresponding 95% confidence intervals. The variance for each estimate was calculated as the square of the standard error. The pooled beta estimate was then computed as a weighted mean, where the weight for each cohort was the inverse of its variance. A 95% confidence interval for the pooled estimate was obtained using the standard error of the weighted mean. The meta-analyses were performed using the metafor package in R (version 4.8-0).

To confirm our EWAS findings, we examined whether the epigenome-wide significant CpGs identified in the discovery cohort (*P* value < 5.95 × 10⁻⁸) were replicated (*P* value < 0.05) in the EPIC-Potsdam cohort. CpGs were classified into three replication categories: Yes, for CpGs reaching nominal significance (*P* < 0.05) with consistent effect direction; Partially, for CpGs with consistent effect directions but *P* > 0.05; and No, for CpGs without consistent effect directions. This discovery-replication framework, in which a stringent threshold is applied in discovery, and nominal significance with directional consistency is required for replication, is an established approach in EWAS that balances type I error control with statistical power^27,28,62,63^. In addition, we evaluated the consistency of the associations by conducting a beta-beta correlation analysis between the beta estimates across all analyzed CpG sites in the two cohorts. Beta-beta plots and corresponding density plots were generated to visually inspect both the concordance of effect sizes and the overall distribution of beta estimates across cohorts. Furthermore, we specifically visualized the beta-beta correlations for the CpGs that reached epigenome-wide significance in the discovery cohort.

### Cross-omics functional analyses of diet-associated DNA methylation

In the discovery cohort, we explored the biological mechanisms underlying the effects of diet on the epigenome by examining diet-associated CpGs for enrichment in gene sets from the Gene Ontology Database (GO) and KEGG pathway database using missMethyl R package (version 1.6.2). The analyses were performed for all diet-associated CpGs.

To explore whether the differential CpGs were associated with gene expression level, we explored expression quantitative trait methylation (eQTM) from the European blood-based BIOS QTL browser (https://molgenis26.gcc.rug.nl/downloads/biosqtlbrowser/) from BBMRI-NL. We further assessed whether the identified CpGs (independent variable) have an impact on their mapped gene expression levels in blood (dependent variable) using data from 1923 participants of the Rhineland Study for whom both methylation data and gene expression data were available. Linear regression models were adjusted for age, sex, batch effects, blood cell proportions, the first ten genetic PCs, and smoking status. As a sensitivity analysis, we further assessed the associations between different diets and the genes mapped to diet-related CpGs.

We used mQTLdb^64^ to examine whether there were previously identified genetic variants, the methylation quantitative trait loci (mQTLs), for the CpGs that were found to be associated with diet quality scores. To evaluate whether the identified CpGs are more likely to be genetically regulated than expected by chance, we additionally performed enrichment analyses using Fisher’s exact tests, restricting both EWAS-identified CpGs and background CpGs to those present on the Illumina 450 K array and tested in our EWAS (conducted on the 850 K array), as currently available mQTL databases are primarily based on the 450 K platform.

To identify whether the differential CpGs were associated with other traits, we looked up CpGs showing associations with the diet quality scores at an epigenome-wide significance level using the EWAS Catalog (http://ewascatalog.org/). We also performed a look-up of known associations of the mapped gene for each CpG in previously published GWAS using the GWAS catalog (https://www.ebi.ac.uk/gwas).

### Reporting summary

Further information on research design is available in the Nature Portfolio Reporting Summary linked to this article.

## Supplementary information


Supplementary Information
Description of Additional Supplementary Files
Supplementary Data
Reporting Summary
Transparent Peer Review file


## Data Availability

Summary-level data supporting the findings of this study are included in the manuscript and its supplementary materials. The complete EWAS summary statistics and analysis scripts are available via Zenodo (https://doi.org/10.5281/zenodo.20511424). The individual-level data from the Rhineland Study and EPIC-Potsdam analyzed in this study are not publicly available because they contain information that could compromise participant privacy and are subject to data protection regulations, including the General Data Protection Regulation (GDPR). Access to the Rhineland Study data can be granted to researchers for academic, non-commercial research purposes, in line with the study’s Data Use and Access Policy (https://www.rheinland-studie.de/en/participation/rules-of-use/). Requests should be submitted to the Rhineland Study Data Use and Access Committee (RS-DUAC@dzne.de), which reviews applications within 4 weeks; access requires a completed data transfer agreement and remains available for the agreed duration of the approved project. Information on access to the EPIC-Potsdam data is available at https://www.dife.de/en/research/cooperations/epic-study/. EPIC-Potsdam data can likewise be obtained for academic, non-commercial research purposes by submitting a formal request to the Principal Investigator of the EPIC-Potsdam study (Prof. Dr. Matthias Schulze; mschulze@dife.de); requests are reviewed within 4 weeks, and access may be subject to a data use agreement.

## Code availability

The analysis scripts are available via Zenodo (https://doi.org/10.5281/zenodo.20511424).
