# Supplementary material for: Associations between diet quality, epigenetic aging and epigenome in two population-based cohorts
Source: Nat Commun. 2026 Aug 29;17:9232. doi: 10.1038/s41467-026-77064-4 (PMC13526023; doi:10.1038/s41467-026-77064-4)
Supplement: Supplementary file 2 [file 41467_2026_77064_MOESM2_ESM.pdf]

Description of Additional Supplementary Files

Supplementary Data 1: Comparison between Rhineland Study included and excluded participants

Supplementary Data 2: Pairwise Pearson correlations between diet quality scores in the Rhineland Study

Supplementary Data 3: Pairwise Pearson correlations between epigenetic aging outcomes in the Rhineland Study

Supplementary Data 4: Association between recommendation-based diet quality scores and epigenetic ageing metrics in the Rhineland Study

Supplementary Data 5: Association between recommendation-based diet quality scores quartiles and epigenetic ageing metrics in the Rhineland Study

Supplementary Data 6: Association between recommendation-based diet quality scores and epigenetic ageing metrics in the Rhineland Study stratified by sex

Supplementary Data 7: Association between recommendation-based diet quality scores and epigenetic ageing metrics in the Rhineland Study stratified by body mass index

Supplementary Data 8: Association between recommendation-based diet quality scores and epigenetic ageing metrics in the Rhineland Study stratified by smoking status

Supplementary Data 9: Numbers of CpGs with epigenome-wide significance based on Bonferroni threshold adjusted p values for each recommendation-based diet quality scores in the Rhineland Study

Supplementary Data 10: Epigenome-wide significant CpG sites associated with diet quality scores in the Rhineland Study

Supplementary Data 11: Overview of the EPIC-Potsdam population characteristics

Supplementary Data 12: Association between recommendation-based diet quality scores and second-generation epigenetic age acceleration metrics in the EPIC-Potsdam

Supplementary Data 13: Pooled meta-analysis estimates for associations between recommendation-based diet quality scores and second-generation epigenetic age acceleration metrics across the Rhineland Study and EPIC-Potsdam

Supplementary Data 14: Association between recommendation-based diet quality scores and second-generation epigenetic age acceleration metrics in the EPIC-Potsdam stratified by sex

Supplementary Data 15: Association between recommendation-based diet quality scores and second-generation epigenetic age acceleration metrics in the EPIC-Potsdam stratified by body mass index

Supplementary Data 16: Association between recommendation-based diet quality scores and second-generation epigenetic age acceleration metrics in the EPIC-Potsdam stratified by smoking status

Supplementary Data 17: Replication status of epigenome-wide significant CpG sites associated with diet quality scores

Supplementary Data 18: CpG overlap among diet quality scores at epigenome-wide significance in the Rhineland Study

Supplementary Data 19: Mapped gene overlap among diet quality scores at epigenome-wide significance in the Rhineland Study

Supplementary Data 20. KEGG pathway overlap among diet quality scores

Supplementary Data 21. GO pathway overlap among diet quality scores

Supplementary Data 22: Overlap of EWAS traits previously associated with CpGs at epigenome-wide significance

Supplementary Data 23:   Overlap of GWAS traits previously associated with identified mapped genes at epigenome-wide significance

Supplementary Data 24: Associations between diet-associated CpGs and the expression levels of their mapped genes in the Rhineland Study

Supplementary Data 25: Enrichment of EWAS-identified CpGs for known mQTLs across diet quality scores

Supplementary Data 26: Distribution of diet quality score quartiles by smoking status in the analytical population in the Rhineland Study.
