# Supplementary material for: Associations between diet quality, epigenetic aging and epigenome in two population-based cohorts
Source: Nat Commun. 2026 Aug 29;17:9232. doi: 10.1038/s41467-026-77064-4 (PMC13526023; doi:10.1038/s41467-026-77064-4)
Supplement: Supplementary file 3 [file 41467_2026_77064_MOESM4_ESM.pdf]

nature portfolio

Corresponding author(s): Monique M.B. Breteler

Last updated by author(s): June 25, 2026

Reporting Summary

Nature Portfolio wishes to improve the reproducibility of the work that we publish. This form provides structure for consistency and transparency in reporting. For further information on Nature Portfolio policies, see our Editorial Policies and the Editorial Policy Checklist.

Statistics

For all statistical analyses, confirm that the following items are present in the figure legend, table legend, main text, or Methods section.

n/a	Confirmed	
☐	☒	The exact sample size (*n*) for each experimental group/condition, given as a discrete number and unit of measurement
☐	☒	A statement on whether measurements were taken from distinct samples or whether the same sample was measured repeatedly
☐	☒	The statistical test(s) used AND whether they are one- or two-sided<br>*Only common tests should be described solely by name; describe more complex techniques in the Methods section.*
☐	☒	A description of all covariates tested
☐	☒	A description of any assumptions or corrections, such as tests of normality and adjustment for multiple comparisons
☐	☒	A full description of the statistical parameters including central tendency (e.g. means) or other basic estimates (e.g. regression coefficient) AND variation (e.g. standard deviation) or associated estimates of uncertainty (e.g. confidence intervals)
☐	☒	For null hypothesis testing, the test statistic (e.g. *F*, *t*, *r*) with confidence intervals, effect sizes, degrees of freedom and *P* value noted<br>*Give P values as exact values whenever suitable.*
☐	☒	For Bayesian analysis, information on the choice of priors and Markov chain Monte Carlo settings
☐	☒	For hierarchical and complex designs, identification of the appropriate level for tests and full reporting of outcomes
☐	☒	Estimates of effect sizes (e.g. Cohen's *d*, Pearson's *r*), indicating how they were calculated

Our web collection on statistics for biologists contains articles on many of the points above.

Software and code

Policy information about availability of computer code

Data collection	No custom computer code was used for data collection.
Data analysis	Statistical analyses were conducted using R (version 4.2.1, The R Foundation) with the following packages: linear regression analyses were performed using the base R lm() function; the tidyverse and broom packages were used to extract model outputs; metafor was used for meta-analyses; missMethyl for gene set enrichment analysis; IlluminaHumanMethylationEPICanno.ilm10b4.hg19, QCEWAS and CMplot for plotting epigenome-wide association study results; and DunedinPACE to calculate DunedinPACE.

For manuscripts utilizing custom algorithms or software that are central to the research but not yet described in published literature, software must be made available to editors and reviewers. We strongly encourage code deposition in a community repository (e.g. GitHub). See the Nature Portfolio guidelines for submitting code & software for further information.

Data

Policy information about availability of data

All manuscripts must include a data availability statement. This statement should provide the following information, where applicable:

- Accession codes, unique identifiers, or web links for publicly available datasets
- A description of any restrictions on data availability
- For clinical datasets or third party data, please ensure that the statement adheres to our policy

Summary-level data supporting the findings of this study are included in the manuscript and its supplementary materials. The individual-level data from the

Rhineland Study and EPIC-Potsdam analyzed in this study are not publicly available because they contain information that could compromise participant privacy and are subject to data protection regulations, including the General Data Protection Regulation (GDPR). Access to the Rhineland Study data can be granted to researchers for academic, non-commercial research purposes, in line with the study's Data Use and Access Policy (https://www.rheinland-studie.de/en/participation/rules-of-use/). Requests should be submitted to the Rhineland Study Data Use and Access Committee (RS-DUAC@dzne.de), which reviews applications within four weeks; access requires a completed data transfer agreement and remains available for the agreed duration of the approved project. Information on access to the EPIC-Potsdam data is available at https://www.dife.de/en/research/cooperations/epic-study/. EPIC-Potsdam data can likewise be obtained for academic, non-commercial research purposes by submitting a formal request to the Principal Investigator of the EPIC-Potsdam study (Prof. Dr. Matthias Schulze; mschulze@dife.de); requests are reviewed within four weeks and access may be subject to a data use agreement. All authors had full access to all data in the study and take responsibility for the integrity of the data and the accuracy of the data analysis.

Research involving human participants, their data, or biological material

Policy information about studies with human participants or human data. See also policy information about sex, gender (identity/presentation), and sexual orientation and race, ethnicity and racism.

Reporting on sex and gender	Of the 6,470 Rhineland Study participants in our analytic sample, 3,665 (57%) were women. Of the 1,034 participants in the EPIC-Potsdam cohort, 640 (62%) were women. Sex-stratified analyses were performed in the current study and showed similar associations between diet quality and epigenetic aging metrics in both sexes. The study analysed biological sex; in the Rhineland Study and EPIC- Potsdam, sex was self-reported and verified against genetic data.
Reporting on race, ethnicity, or other socially relevant groupings	Our analyses were based on data from participants of European ancestry from two independent cohorts (i.e., the Rhineland Study and the EPIC-Potsdam study). The analyses were restricted to individuals of European ancestry, as they constituted the majority of participants in both cohorts.
Population characteristics	Participants' mean age (± standard deviation) was 56.3 ± 13.6 years in the Rhineland Study and 49.9 ± 8.9 years in the EPIC-Potsdam cohort. Information on sex and ethnicity is provided above.
Recruitment	The Rhineland Study is an ongoing, population-based prospective cohort study. It recruits individuals aged 30 years or older who reside in one of two municipal districts in Bonn, Germany, and who have sufficient command of the German language to provide informed consent. A primary objective of the Rhineland Study is to identify determinants and markers of healthy ageing through a deep-phenotyping approach. At baseline, participants undergo an 8-hour, in-depth, multi-domain phenotypic assessment, and various types of biomaterials (including blood, urine, stool, and hair) are collected.<br>The EPIC-Potsdam study is a prospective cohort study based in Germany and part of the larger European Prospective Investigation into Cancer and Nutrition (EPIC) multicenter study. It includes 27,548 men and women, primarily aged 35–65 years, who were recruited between 1994 and 1998. Participants were selected from the general population of Potsdam and the surrounding area and were invited to a baseline examination conducted by trained staff. At baseline, computer-assisted interviews on lifestyle and medical history were conducted. Follow-up questionnaires have been mailed to participants every 2–3 years to assess incident disease cases.
Ethics oversight	The Rhineland Study was approved by the Ethics Committee of the Medical Faculty at the University of Bonn and is conducted in accordance with the principles of the Declaration of Helsinki. The EPIC-Potsdam study was approved by the Ethics Committee of the Federal State of Brandenburg, and written informed consent was obtained from all participants at the time of recruitment.

Note that full information on the approval of the study protocol must also be provided in the manuscript.

Field-specific reporting

Please select the one below that is the best fit for your research. If you are not sure, read the appropriate sections before making your selection.

☒ Life sciences ☐ Behavioural & social sciences ☐ Ecological, evolutionary & environmental sciences

For a reference copy of the document with all sections, see nature.com/documents/nr-reporting-summary-flat.pdf

Life sciences study design

All studies must disclose on these points even when the disclosure is negative.

Sample size	We included 6,470 participants from the Rhineland Study and 1,034 participants from the EPIC-Potsdam study.
Data exclusions	This study is based on participants with complete information on diet, epigenetic data, and relevant covariates.
Replication	To validate the results from the Rhineland Study, we conducted a replication analysis in an independent cohort, the EPIC-Potsdam study. This study employed similar methods for measuring dietary intake and DNA methylation levels as those used in the Rhineland Study. Overall, the associations between diet quality scores and epigenetic age acceleration metrics in EPIC-Potsdam closely mirrored those observed in the Rhineland Study, both in direction and magnitude. Of the 240 epigenome-wide significant CpGs identified in the Rhineland Study across nine dietary patterns, 39 were successfully replicated with consistent effect directions. Furthermore, even among CpGs that did not reach nominal significance in the replication cohort, more than 50% of the top CpGs retained consistent effect directions with the discovery cohort. Beta-beta correlation analyses of the epigenome-wide significant CpGs further supported the consistency of findings between the Rhineland Study and EPIC-Potsdam.

Randomization	N/A
Blinding	N/A

Reporting for specific materials, systems and methods

We require information from authors about some types of materials, experimental systems and methods used in many studies. Here, indicate whether each material, system or method listed is relevant to your study. If you are not sure if a list item applies to your research, read the appropriate section before selecting a response.

Materials & experimental systems

n/a	Involved in the study	
☒	☐	Antibodies
☒	☐	Eukaryotic cell lines
☒	☐	Palaeontology and archaeology
☒	☐	Animals and other organisms
☒	☐	Clinical data
☒	☐	Dual use research of concern
☒	☐	Plants

Methods

n/a	Involved in the study	
☒	☐	ChIP-seq
☒	☐	Flow cytometry
☒	☐	MRI-based neuroimaging

Plants

Seed stocks	*Report on the source of all seed stocks or other plant material used. If applicable, state the seed stock centre and catalogue number. If plant specimens were collected from the field, describe the collection location, date and sampling procedures.*
Novel plant genotypes	*Describe the methods by which all novel plant genotypes were produced. This includes those generated by transgenic approaches, gene editing, chemical/radiation-based mutagenesis and hybridization. For transgenic lines, describe the transformation method, the number of independent lines analyzed and the generation upon which experiments were performed. For gene-edited lines, describe the editor used, the endogenous sequence targeted for editing, the targeting guide RNA sequence (if applicable) and how the editor was applied.*
Authentication	*Describe any authentication procedures for each seed stock used or novel genotype generated. Describe any experiments used to assess the effect of a mutation and, where applicable, how potential secondary effects (e.g. second site T-DNA insertions, mosiacism, off-target gene editing) were examined.*
