# Supplementary material for: Associations between diet quality, epigenetic aging and epigenome in two population-based cohorts
Source: Nat Commun. 2026 Aug 29;17:9232. doi: 10.1038/s41467-026-77064-4 (PMC13526023; doi:10.1038/s41467-026-77064-4)
Supplement: Supplementary file 4 [file 41467_2026_77064_MOESM5_ESM.pdf]

nature portfolio

Peer Review File

Associations between diet quality, epigenetic aging and epigenome: Findings from two population-based Studies

Corresponding Author: Professor Monique Breteler

This file contains all reviewer reports in order by version, followed by all author rebuttals in order by version.

Version 1:

Reviewer comments:

Reviewer #2

(Remarks to the Author)
This paper reports on a creative and ambitious study that leverages dietary and epigenetic data from two large, population-based European cohort studies. Key strengths include an extensive set of analyses that consider dietary exposures to gene expression, use of validated food frequency questionnaires and classification of dietary patterns as opposed to single foods or nutrients, with dietary patterns scored based on established links to disease prevention. An especially interesting finding is the convergence of biological pathways across different dietary patterns, suggesting shared mechanisms of effect as well as the alignment of biological pathways targeted by various dietary patterns and ultimately affected (i.e. DASH on pathways related to cardiovascular functioning etc.).

However, the paper would benefit from additional clarification and detail in several areas. The authors should account for cardiometabolic conditions beyond obesity (e.g., diabetes, hypertension) as potential confounders or include sensitivity analyses addressing them. More information is needed on epigenetic clock generation, including handling of missing CpGs. Several associations are reported as significant without appropriate multiple testing corrections (e.g., Figure 2 and similar analyses), and key figures and supplements lack test statistics such as beta coefficients or p-values.

Key methodological details need clarification, including how dietary pattern overlap was defined, FFQ completeness thresholds, and data collection timing across cohorts. The authors should also explain the ancestry composition of both cohorts and why non-Caucasian participants were excluded. Finally, the discussion should more evenly address both similarities and differences across dietary patterns and cohorts, rather than focusing solely on convergence.

Major concerns:

Multiple Comparison Adjustments – Bonferroni adjustments are used to address multiple comparisons for the genome-wide analyses but not other analyses. Please make sure you have adjusted for multiple comparisons throughout all your analyses (i.e. throughout all the age acceleration and phenotype tests). For example Figure 2a reports results from 20 tests without adjusting the significance level. The confidence intervals reported in 2a do not reflect the multiple tests. Please do not report results as “significant” here without considering multiple testing adjustment.
Tables & Figures - Some key test statistics are missing from the presentation of your results. For example, Table S2 appropriately shows p-values from the quantile-based sensitivity analysis, but additional statistics (beta/SD and/or T-statistics) would be helpful. More importantly, there is no equivalent table for the main analysis that is presented in Figure 2. Since significance is being reported for this figure, relevant test statistics and p-values should be presented.

Other concerns:

Abstract
Line 49 – What does “independent” refer to here?
Line 50 – Minimal overlap of participants in the top 25% of adherence means that the people who were most adherent to one dietary pattern were not adherent to other patterns?
Line 53 – It is implied that these are beneficial pathways but what if harm is caused?
Lines 54-56 – “Indicates” seems stronger than warranted.
Introduction

Line 80 – The concept of overlap is key to the analyses conducted in this study. This paragraph could use a single sentence or two clarifying what is meant by overlap. From what I can tell, you're referring to the idea that individuals may be adhering (to some degree) to multiple healthy dietary patterns simultaneously.

Methods
Line 368/Supplemental Figure 1– Why were participants of non-Caucasian descent removed (702)? Why did about 1000 have missing or doubtful FFQ data? (Supplemental Figure 1)?
Line 376 – Does this mean that participants needed to have responses for at least 80% of the food items in the FFQ food list?
Line 376 – Were the FFQ measures collected during the same time point as the buffy coat samples for both Rhineland and EPIC-Potsdam?
Line 393 – What R package was used to generate the clocks?
Line 393 – Most clocks were developed on the 450k array. Were there any clock CpG sites missing on the 850k array? If so, what did you do about this?
Line 403 – Prevalent cardiometabolic disease may be another confounder of dietary pattern and epigenetic age. Individuals with prevalent disease may be following specific dietary patterns because of this. Prevalent cardiometabolic disease (diabetes, hypertension) is associated with accelerated aging. You account for BMI, but there are metabolically healthy individuals who are overweight, and metabolically unhealthy individuals at normal BMIs. Your effect estimates were slightly attenuated when including BMI in the model, therefore may be worth considering cardiometabolic diseases.
Line 421 – Why did you select these clocks vs. first and third generation?
Line 424 – Please explain more about the adjustment for batch effects that was performed. Why not chip? Row?
Line 460 – Specify EPIC v1.0 now that there are multiple versions.

Results
Line 168 – Consider including the age range for EPIC – Potsdam in parentheses as you did for Rhineland.
Line 168 – Why only 1k people from EPIC Potsdam? Was this not a much larger study? Who was included?
Line 183 – "significant differences" are described but no test statistics are presented. These results should be reported on the figure (Supp Fig 8) or in a supplementary table.
Lines 244-249: it is mentioned that several CpGs associated with mQTLs, but no information is provided about these in the text or a supplemental table, which makes this statement hard to interpret. For CpGs that are present on the 450K array, it would be possible to perform an enrichment test of whether more mQTL-associated CpGs are detected than expected by chance. (A similar test could also be helpful to help interpret the eQTM results.)
As mentioned above, 20 tests are performed in Figure 2, with many reported as "significant", but no multiple testing appears to be applied.
Discussion
Line 254 – How many people were smokers and adhered to any of the healthy dietary patterns?
Line 282 – Here you discuss the similarities in findings between the training and validation cohorts, but how do you make sense of the differences in what you found between the two?
Line 333 – Based on inclusion criteria, specify German population of Caucasian ancestry. What is the ancestry of the EPIC-Potsdam cohort?

Tables & Figures
Table 1 - Here and in other relevant descriptive tables:
- Consider including a descriptor of socioeconomic status in these tables as this is another potential confounder.
- Include a footnote that describes how high/medium/low education are classified
- Consider including prevalence of other cardiometabolic diseases aside from obesity (i.e. diabetes, hypertension)
- Are you reporting the mean residual value for GrimAA and PhenoAA? What were the actual GrimAge and PhenoAge values?
Supplementary Tables 11 and 12 lack important information such as citations to the previous studies indicated, as well as the cg names involved.
In addition to Supplementary Figure 7, scatterplots like Supplementary Figure 11 (but with just one point per measure) could be informative to show the correspondence between the effect sizes across discovery (Figure 2) and replication (Supp Fig 7) for the different measures.
Supplementary Figure 11 – Correlation should be reported separately by measure; reporting correlation of effect sizes for all measures combined is likely to be misleading, especially given the differences in scale seen in the figure.
Supplementary Table 10 – "Pathways" is misspelled. Please specify what columns DE and P.DE stand for.
Supplementary Figures 15 and 16 – What does FDR refer to in the legend? Assuming FDR correction. Please describe in footnote.

Reviewer #3

(Remarks to the Author)
I co-reviewed this manuscript with one of the reviewers who provided the listed reports. This is part of the Nature Communications initiative to facilitate training in peer review and to provide appropriate recognition for Early Career Researchers who co-review manuscripts.

Reviewer #4

(Remarks to the Author)
The authors performed a series of elegant analyses examining the associations of 10 different diet quality scores with markers of accelerated epigenetic aging and DNA methylation profiles. The discovery sample was derived from the Rhineland Study, a population-based study of individuals aged 30 years or older. The authors sought replication of their findings in participants from the EPIC-Potsdam study.
The study reports associations between higher "healthy" dietary pattern adherence scores (except for the EAT-Lancet score) and lower epigenetics indices of biological age, and between higher "unhealthy" dietary pattern adherence scores and higher epigenetic indices of biological age. Stratified analyses revealed that these associations were driven (or were generally stronger) amongst current smokers compared to former and never smokers. In EWAS analyses, except for the EAT-Lancet score, diet scores exhibited epigenome-wide significant associations with DNA methylation sites, with mostly negative beta coefficients for 'healthy" dietary patterns and positive beta coefficients for "unhealthy" dietary patterns.
This work provides valuable insights into the potential influences of diet on biological aging. Nevertheless, there are certain methodological issues that warrant further attention; these are outlined below.
The authors should consider adjusting the associations between diet quality scores and markers of accelerated epigenetic aging for physical activity (at least as a sensitivity analysis). It is possible that people with higher diet quality scores also maintain an overall healthier lifestyle and are engaged in regular physical exercise. Physical activity has been associated with slower epigenetic aging, therefore, potentially acting as a confounder in the reported associations.

The authors should consider presenting the beta coefficients and the respective confidence intervals of the reported associations between higher diet quality scores and epigenetic aging indices in a table format or in the narrative. It is rather difficult for the reader to determine from Figure 2 which associations are significant since, for some associations such as those of the MDS, MIND, and PDI with GrimAA it appears as the confidence interval band intercepts the null (horizontal) line.

It is somewhat unclear why in the replication cohort the nominal threshold for statistical significance ($p < 0.05$) was used for EWAS analyses instead of the epigenome-wide significance level ($p < 5.95 \times 10^{-8}$). A potentially more robust replication approach would include EWAS studies being conducted in parallel in the two independent cohorts (without using the findings in the discovery cohort to inform replication), epigenome-wide significant associations being determined independently, and then assessing the overlap of the identified associations between the two cohorts?

It is somewhat unclear why the GrimAA and PhenoAA epigenetic age acceleration metrics were selected compared to other measures. For example, a recent study that tested whether the pace of biological aging mediates the relationships between adherence to a healthy diet and incident dementia and mortality, used the DunedinPACEepigenetic clock to measure the pace of biological aging (PMID: 38407506). This index has the advantage that it was derived from 19 biomarkers ascertained longitudinally, preventing short-term illnesses from affecting aging measurements, and capturing gradual, long-term changes that truly reflect aging. The authors should consider further justifying their selection of epigenetic metrics, describe potential advantages and pitfalls compared to other measures that led them to this selection, and outline potential differences in the aspects of biological aging each measure captures (i.e., how are these measures similar and how they differ).

The authors evaluated potential effect modification of the diet quality scores by sex, smoking status, and BMI on epigenetic age acceleration. What were the generating hypotheses behind these analyses? The authors should consider elaborating on that a bit more.

In Supplemental Table 6 where the authors describe the results of the replication analysis of the EWAS study, it is somewhat unclear what "Partial" under the "Replicated" column means.

A potential limitation the authors should consider pointing out pertains to the generalizability of their findings in different race/ethnicities. There is evidence to suggest that the performance of DNA methylation-based predictors of chronological age (i.e., epigenetic clocks) is affected by race and ethnicity (PMID: 40205465). Therefore, the generalizability and external validity of the present findings to other racial/ethnic groups cannot be ascertained within the context of this study and needs to be further assessed.

In gene expression analyses, only 33 diet-associated CpGs were associated with their mapped gene expression levels. The authors should consider providing some tentative explanations on how identified associations between diet and the remaining DNA methylation sites (i.e. those not being associated with mapped gene expression levels), might be biologically relevant

Reviewer #5

(Remarks to the Author)
I co-reviewed this manuscript with one of the reviewers who provided the listed reports. This is part of the Nature Communications initiative to facilitate training in peer review and to provide appropriate recognition for Early Career Researchers who co-review manuscripts.

Version 2:

Reviewer comments:

Reviewer #2

(Remarks to the Author)
For the most part the authors have done an excellent job of addressing our revisions. Thank you for carefully addressing our points and adding enrichment analyses, sensitivity analyses, multiple testing adjustment, supplemental data, and clarification where requested. Below we raise a few minor and easily addressable concerns, mostly related to 1) avoiding overstatement in the abstract/introduction, 2) comparisons to previous results, and 3) needed clarifications in the text of the manuscript.

1) Avoid overstatement and unsupported statements in the abstract and the introduction
- The first sentence of the abstract is misleading in that it seems to suggest DNA methylation changes as a prime candidate to mediate the relationship between diet and health, when really the evidence presented for this is quite limited. Perhaps this can be toned down or removed.
- The final sentence of the abstract is improved but probably still stronger than warranted since a causal connection to health outcomes has not been established. How about the addition of the word "could" so that it reads “suggests that general adherence to a healthy dietary pattern could promote health”?
- Refs 28 and 29 are described as showing evidence that “diet quality can significantly impact an individual’s epigenome”. However, the beta coefficients from associations reported in these papers are quite small, with only a few replicating across cohorts, suggesting subtle rather than significantly impactful changes. This wording could be toned down.
- It is unclear why ref 26 was cited; the topic and finding does not seem very related to the statement it is supposed to support. And neither reference 26 nor 27 supports use of the word “crucial” here.
- The rest of the introduction seems to state findings accurately without overstatement.

2) Comparison to previous results
- Would be useful to indicate what “SeqId” refers to in header of Supplemental Table 8
- Would be useful to mention in Results that you’re using the EWAS catalog to identify previous results; this will save the reader for having to hunt this down in Methods.
- Although some of this information may be in SuppTab 8 and mixed in with the other results, it would be very useful to separately examine (and describe in the text) whether any of your replicated EWAS CpGs overlap with CpGs reported in refs 28 or 29.
- Supplemental Table 8 is referenced twice in the text; for the second reference I believe you intended to reference Supplemental Table 21.

3) Points satisfactorily addressed in response but requiring further clarification in paper. Here we agreed with the response but wanted to see further clarification of these points in the text. Here we have reprinted the original responses, with our recommendation for each point listed after "REVIEWER RESPONSE".

Line 368/Supplemental Figure 1– Why were participants of non-Caucasian descent removed (702)? Why did about 1000 have missing or doubtful FFQ data? (Supplemental Figure 1)?

We restricted analyses to participants of European ancestry to minimize confounding by ancestry-related differences in DNA methylation patterns and to ensure comparability with epigenetic clocks and EWAS reference resources, which have been predominantly developed and calibrated in European-ancestry populations. In addition, both the Rhineland Study and the EPIC-Potsdam participants are predominantly German of Caucasian descent. Given the relatively small number of non-European ancestry participants with complete data, we were not able to perform robust ancestry-stratified analyses.

The missing dietary assessment (n=841) cases were participants who did not complete the FFQ at baseline (i.e., no dietary questionnaire data were available). Following the transition to the web-based format, the FFQ was completed predominantly at home via an emailed link. Participants who reported being unable to complete it at home were offered an additional appointment at the study center. Nevertheless, because completion occurred outside the study center for most participants, we cannot infer specific reasons for non-completion.

Among participants with baseline dietary assessment, incomplete or doubtful FFQ data (n=200) reflects questionnaires that did not meet our predefined quality criteria. Specifically, n=197 did not complete at least 80% of FFQ items (the completion threshold we have adopted, similarly to previous studies)1, and n=3 were excluded during quality assurance due to concerns about questionnaire validity (e.g., insufficient German language proficiency flagged by study staff). We updated the flowchart of included participants to reflect this distinction (Supplementary Figure S1).

Reference:
1. Knüppel, S., Clemens, M., Conrad, J. et al. Design and characterization of dietary assessment in the German National Cohort. Eur J Clin Nutr 73, 1480–1491 (2019). https://doi.org/10.1038/s41430-018-0383-8

REVIEWER RESPONSE: Satisfactory explanation. At a minimum, please include footnotes to Supplementary Figure 1 addressing these points. If there is space, please include at least one sentence addressing each point in the manuscript methods.

Line 376 – Does this mean that participants needed to have responses for at least 80% of the food items in the FFQ food list?

Yes. For inclusion in the analyses, participants were required to provide responses for at least 80% of FFQ food and

beverage items. FFQs below this completeness threshold were considered incomplete and excluded1 (corresponding to the n=197 exclusions shown in Supplementary Figure 1).

Reference:

1. Knüppel, S., Clemens, M., Conrad, J. et al. Design and characterization of dietary assessment in the German National Cohort. Eur J Clin Nutr 73, 1480–1491 (2019). https://doi.org/10.1038/s41430-018-0383-8

REVIEWER RESPONSE: Satisfactory explanation. Please also include a sentence or two specifying this in the manuscript.

Line 376 – Were the FFQ measures collected during the same time point as the buffy coat samples for both Rhineland and EPIC-Potsdam?

In both the Rhineland Study and EPIC-Potsdam, the FFQ and buffy coat samples were collected at the same time during the baseline assessment.

REVIEWER RESPONSE: Satisfactory explanation. Please also specify in the manuscript.

Line 168 – Why only 1k people from EPIC Potsdam? Was this not a much larger study? Who was included?

The EPIC-Potsdam study is indeed a large population-based cohort with approximately 27,000 participants.1 DNA methylation was measured using the Illumina MethylationEPIC v1.0 array in a randomly selected subcohort of EPIC-Potsdam (n= 1147) as part of a nested case-cohort design. 2 The 1,034 participants included in our replication analysis represent those with available EPIC v1.0 array methylation data and complete dietary and covariate information at the time of our analysis. This subsample is broadly representative of the overall EPIC-Potsdam cohort in terms of key demographic characteristics. The sample size is consistent with other recent methylation studies from EPIC-Potsdam2 and provides adequate statistical power for replication of epigenome-wide findings, as demonstrated by our successful validation of associations for diet quality scores with epigenetic aging markers and CpG sites.

References:

1. Boeing H, et al. Recruitment procedures of EPIC-Germany. Ann Nutr Metab. 1999;43(4):205-15.
2. Eroglu, B., Eichelmann, F., Kuxhaus, O. et al. Trace element-linked DNA methylation sites and their association with type 2 diabetes and cardiovascular diseases: EPIC-Potsdam cohort study. Clin Epigenet 17, 172 (2025).

REVIEWER RESPONSE: Were the sub-sample characteristics comparable to the overall study population? Selection bias among those who had methylation data?

Reviewer #3

(Remarks to the Author)
I co-reviewed this manuscript with one of the reviewers who provided the listed reports. This is part of the Nature Communications initiative to facilitate training in peer review and to provide appropriate recognition for Early Career Researchers who co-review manuscripts.

Reviewer #4

(Remarks to the Author)

Reviewer #5

(Remarks to the Author)
I co-reviewed this manuscript with one of the reviewers who provided the listed reports. This is part of the Nature Communications initiative to facilitate training in peer review and to provide appropriate recognition for Early Career Researchers who co-review manuscripts.

Open Access This Peer Review File is licensed under a Creative Commons Attribution 4.0 International License, which permits use, sharing, adaptation, distribution and reproduction in any medium or format, as long as you give appropriate credit to the original author(s) and the source, provide a link to the Creative Commons license, and indicate if changes were made.
In cases where reviewers are anonymous, credit should be given to 'Anonymous Referee' and the source.
The images or other third party material in this Peer Review File are included in the article's Creative Commons license, unless indicated otherwise in a credit line to the material. If material is not included in the article's Creative Commons license and your intended use is not permitted by statutory regulation or exceeds the permitted use, you will need to obtain permission directly from the copyright holder.
To view a copy of this license, visit https://creativecommons.org/licenses/by/4.0/

REVIEWER COMMENTS

Reviewer #2 (Remarks to the Author):

This paper reports on a creative and ambitious study that leverages dietary and epigenetic data from two large, population-based European cohort studies. Key strengths include an extensive set of analyses that consider dietary exposures to gene expression, use of validated food frequency questionnaires and classification of dietary patterns as opposed to single foods or nutrients, with dietary patterns scored based on established links to disease prevention. An especially interesting finding is the convergence of biological pathways across different dietary patterns, suggesting shared mechanisms of effect as well as the alignment of biological pathways targeted by various dietary patterns and ultimately affected (i.e. DASH on pathways related to cardiovascular functioning etc.).

However, the paper would benefit from additional clarification and detail in several areas. The authors should account for cardiometabolic conditions beyond obesity (e.g., diabetes, hypertension) as potential confounders or include sensitivity analyses addressing them. More information is needed on epigenetic clock generation, including handling of missing CpGs. Several associations are reported as significant without appropriate multiple testing corrections (e.g., Figure 2 and similar analyses), and key figures and supplements lack test statistics such as beta coefficients or p-values.

Key methodological details need clarification, including how dietary pattern overlap was defined, FFQ completeness thresholds, and data collection timing across cohorts. The authors should also explain the ancestry composition of both cohorts and why non-Caucasian participants were excluded. Finally, the discussion should more evenly address both similarities and differences across dietary patterns and cohorts, rather than focusing solely on convergence.

Major concerns:

Multiple Comparison Adjustments – Bonferroni adjustments are used to address multiple comparisons for the genome-wide analyses but not other analyses. Please make sure you have adjusted for multiple comparisons throughout all your analyses (i.e. throughout all the age acceleration and phenotype tests). For example Figure 2a reports results from 20 tests without adjusting the significance level. The confidence intervals reported in 2a do not reflect the multiple tests. Please do not report results as “significant” here without considering multiple testing adjustment.

We appreciate the reviewer's emphasis on multiple-testing control, which strengthens the reliability of our conclusions. In the original submission, we applied Bonferroni correction for the EWAS analyses, which is standard practice given the very large number of CpG tests. For the analyses relating diet quality scores to epigenetic age acceleration, our primary emphasis was on the pattern and consistency of effect estimates across dietary indices rather than on isolated nominally significant p-values (i.e., below 0.05). However, we agree that multiple comparisons should also be addressed for these analyses when results are interpreted as statistically significant (e.g., the set of tests presented in Figure 2a).

Accordingly, in the revised manuscript we now control the false discovery rate using the Benjamini-Hochberg procedure for all analyses relating the diet quality scores to epigenetic age acceleration. We selected BH-FDR to balance type I error control with statistical power in these correlated hypothesis sets. Specifically, BH-FDR correction is applied within each epigenetic age acceleration outcome across the set of diet quality scores tested (i.e., 10 dietary scores per outcome in Figure 2a; 20 tests total across both outcomes). We apply the same approach to the BMI-adjusted sensitivity model and to stratified analyses (smoking, sex, and BMI), with BH-FDR performed within each outcome (e.g., 30 tests per outcome in the smoking-stratified analysis: 10 dietary scores × 3 smoking categories). We now report both nominal p-values and BH-FDR q-values in the Supplementary Tables, and we have revised the figure and manuscript text to avoid describing findings as statistically significant unless they remain significant after multiple-testing adjustment.

<u>Changes in manuscript and supplementary files:</u>

- Methods updated to describe BH-FDR correction for diet quality score-epigenetic age acceleration analyses (within outcome across dietary indices) and to clarify interpretation of statistical significance (page 19, lines 538-542): *"To account for multiple comparisons, we controlled for false discovery rate (FDR) using the Benjamini-Hochberg procedure within each epigenetic age acceleration outcome. Results were considered statistically significant at FDR q < 0.05; nominal p-values are reported for completeness."*
- Figure 2 and corresponding Results text updated so statements of statistical significance are based on BH-FDR q-values (page 6, lines 144-159).
- Supplementary Figures S3-S5 updated so point shapes reflect BH-FDR significance categories.
- Added Supplementary Table S6 providing full regression results underlying Figure 2a and the BMI-adjusted sensitivity model (β, SE, test statistic, 95% CI, nominal p-value, and BH-FDR q-value).
- Added Supplementary Tables S4-S6 providing full regression results for stratified analyses (sex, BMI, smoking), including interaction p-values (Int_P) and BH-FDR–adjusted interaction q-values (Int_FDR).

Tables & Figures - Some key test statistics are missing from the presentation of your results. For example, Table S2 appropriately shows p-values from the quantile-based sensitivity analysis, but additional statistics (beta/SD and/or T-statistics) would be helpful. More importantly, there is no equivalent table for the main analysis that is presented in Figure 2. Since significance is being reported for this figure, relevant test statistics and p-values should be presented.

We agree that including the accompanying model statistics (e.g., standard errors and/or t-statistics and exact p-values), as well as multiple-testing–adjusted values, improves the transparency of these results.

Accordingly, we have updated Supplementary Table S2 to include full regression outputs for the quartile-based analyses, including β estimates, standard errors, t-statistics, 95% confidence intervals, nominal p-values, and BH-FDR-adjusted q-values for Q2-Q4 versus Q1. We also expanded the p-trend reporting to include the corresponding β estimate (based on modeling the quartile-specific median score as a continuous variable), 95% CI, t-statistic, and both nominal and BH-FDR–adjusted p-values. This revised table now fully reflects the model results underlying the quartile sensitivity analyses described in the Methods.

In addition, as noted in our response to the previous comment, we have added supplementary tables providing full regression outputs for the main analyses presented in Figure 2 and for all sensitivity and

stratified analyses (β, SE, test statistics, 95% CIs, nominal p-values, BH-FDR q-values, and interaction p-values where applicable).

Changes in supplementary files:

- Supplementary Table S2 updated to include full regression statistics for quartile-based analyses (β, SE, t-statistic, 95% CI, nominal p-value, and BH-FDR q-value for Q2–Q4 vs Q1), and expanded p-trend reporting (β, 95% CI, t-statistic, nominal p-value, BH-FDR q-value).
- Supplementary Table S3 provides full regression results underlying Figure 2a (Model 1; Table S3 also includes the BMI-adjusted sensitivity Model 2).
- Supplementary Table S6 provides the full regression results underlying Figure 2b (stratified by smoking status), including interaction p-values and BH-FDR–adjusted interaction q-values.
- Supplementary Tables S4-S6 added to provide full regression outputs for stratified analyses, including interaction terms and multiple-testing adjustment: Table S4 (stratified by sex), Table S5 (stratified by BMI category), and Table S6 (stratified by smoking status), each reporting β, SE, test statistics, 95% CIs, nominal p-values, BH-FDR q-values, interaction p-values, and BH-FDR–adjusted interaction q-values.

Other concerns:

Abstract
Line 49 – What does "independent" refer to here?

Here, 'independent' was intended to indicate that EPIC-Potsdam served as an external validation cohort comprising different participants (i.e., no overlap with the Rhineland Study). We have revised the Abstract accordingly: *"We used data from the Rhineland Study, a large population-based cohort, and validated our findings using corresponding data from the EPIC-Potsdam cohort."* (page 3, line 49).

Line 50 – Minimal overlap of participants in the top 25% of adherence means that the people who were most adherent to one dietary pattern were not adherent to other patterns?

To clarify, 'minimal overlap of participants in the top 25% of adherence' refers to overlap in highest-quartile classification across diet quality scores. Specifically, participants classified in the highest quartile (top 25%) for one diet quality score were rarely the same participants classified in the highest quartile for other scores. This does not imply non-adherence to other dietary patterns; rather, individuals may show moderate or relatively high adherence across multiple scores without simultaneously falling within the highest quartile for each. We have revised the Abstract to make this interpretation explicit, changing 'in the top 25%' to *"we found minimal overlap of participants classified in the highest quartile (top 25%) of adherence across different diet quality scores."* (page 3, line 50).

Line 53 – It is implied that these are beneficial pathways but what if harm is caused?

We appreciate the reviewer raising this interpretive point. The phrase 'converged onto the same biological pathways' is a descriptive observation from pathway enrichment analysis and does not itself imply that these pathways are beneficial or harmful. The concluding statement ('promotes health through similar epigenetic mechanisms') is grounded in the observed associations between higher adherence to healthy dietary patterns and lower epigenetic age acceleration, rather than implying that pathway enrichment denotes uniformly beneficial effects or that causality is established. To better reflect the associational nature of our findings, we have tempered the language by replacing 'indicates' with 'suggests' (see response to Lines 54-56).

Lines 54-56 – "Indicates" seems stronger than warranted.

Following the reviewer's suggestion, we have tempered the strength of the concluding statement in the Abstract by replacing 'indicates' with 'suggests' (page 3, line 54).

Introduction

Line 80 – The concept of overlap is key to the analyses conducted in this study. This paragraph could use a single sentence or two clarifying what is meant by overlap. From what I can tell, you're referring to the idea that individuals may be adhering (to some degree) to multiple healthy dietary patterns simultaneously.

To clarify the concept of overlap used throughout the study, we have now added two sentences to the first paragraph of the Introduction (page 4, lines 80–84): *"Because these diet quality scores partially capture shared dietary behaviours, individuals may score highly on more than one pattern at the same time. Although each score emphasizes different dietary components, they share common features, such as higher intake of fruits, vegetables, and whole grains, thus resulting in overlapping representations of overall diet quality."*

Methods

Line 368/Supplemental Figure 1– Why were participants of non-Caucasian descent removed (702)? Why did about 1000 have missing or doubtful FFQ data? (Supplemental Figure 1)?

We restricted analyses to participants of European ancestry to minimize confounding by ancestry-related differences in DNA methylation patterns and to ensure comparability with epigenetic clocks and EWAS reference resources, which have been predominantly developed and calibrated in European-ancestry populations. In addition, both the Rhineland Study and the EPIC-Potsdam participants are predominantly German of Caucasian descent. Given the relatively small number of non-European ancestry participants with complete data, we were not able to perform robust ancestry-stratified analyses.

The missing dietary assessment (n=841) cases were participants who did not complete the FFQ at baseline (i.e., no dietary questionnaire data were available). Following the transition to the web-based format, the FFQ was completed predominantly at home via an emailed link. Participants who reported being unable to complete it at home were offered an additional appointment at the study center. Nevertheless, because completion occurred outside the study center for most participants, we cannot infer specific reasons for non-completion.

Among participants with baseline dietary assessment, incomplete or doubtful FFQ data (n=200) reflects questionnaires that did not meet our predefined quality criteria. Specifically, n=197 did not complete at least 80% of FFQ items (the completion threshold we have adopted, similarly to previous studies)[1], and n=3 were excluded during quality assurance due to concerns about questionnaire validity (e.g., insufficient German language proficiency flagged by study staff). We updated the flowchart of included participants to reflect this distinction (Supplementary Figure S1).

Reference:

1. Knüppel, S., Clemens, M., Conrad, J. et al. Design and characterization of dietary assessment in the German National Cohort. Eur J Clin Nutr 73, 1480–1491 (2019). https://doi.org/10.1038/s41430-018-0383-8

Line 376 – Does this mean that participants needed to have responses for at least 80% of the food items in the FFQ food list?

Yes. For inclusion in the analyses, participants were required to provide responses for at least 80% of FFQ food and beverage items. FFQs below this completeness threshold were considered incomplete and excluded[1] (corresponding to the n=197 exclusions shown in Supplementary Figure 1).

Reference:

1. Knüppel, S., Clemens, M., Conrad, J. et al. Design and characterization of dietary assessment in the German National Cohort. Eur J Clin Nutr 73, 1480–1491 (2019). https://doi.org/10.1038/s41430-018-0383-8

Line 376 – Were the FFQ measures collected during the same time point as the buffy coat samples for both Rhineland and EPIC-Potsdam?

In both the Rhineland Study and EPIC-Potsdam, the FFQ and buffy coat samples were collected at the same time during the baseline assessment.

Line 393 – What R package was used to generate the clocks?

PhenoAge and GrimAge were computed by implementing the published algorithms from Levine et al. and Lu et al.[1,2] using the specified CpG sets (513 and 1030 CpGs, respectively) and the corresponding coefficients; age acceleration was defined as the residual from regressing each DNAm age estimate on chronological age (as described in the Methods; thus no specific R packages were used for generating these clocks). DunedinPACE was calculated using the DunedinPACE R package (https://github.com/danbelsky/DunedinPACE).[3] We have now added the DunedinPACE calculation in the Methods (page 17, line 470-475).

References:

1. Levine ME, et al. An epigenetic biomarker of aging for lifespan and healthspan. Aging (Albany NY) 10, 573-591 (2018).
2. Lu AT, et al. DNA methylation GrimAge strongly predicts lifespan and healthspan. Aging (Albany NY) 11, 303-327 (2019).
3. Belsky DW, Caspi A, Corcoran DL, Sugden K, Poulton R, Arseneault L, Baccarelli A, Chamarti K, Gao X, Hannon E, Harrington HL, Houts R, Kothari M, Kwon D, Mill J, Schwartz J, Vokonas P, Wang C, Williams BS, Moffitt TE. DunedinPACE, a DNA methylation biomarker of the pace of aging. Elife. 2022 Jan 14;11:e73420.

Line 393 – Most clocks were developed on the 450k array. Were there any clock CpG sites missing on the 850k array? If so, what did you do about this?

PhenoAge, GrimAge and DunedinPACE were originally developed using CpG sites that are present on both the Illumina 450K and 850K/EPIC v1 array to ensure the cross-platform compatibility.[1,2,3] Accordingly, all CpG sites required for the calculation of these clocks were available in our EPIC v1 dataset and passed quality control. No clock CpGs were missing, and therefore no imputation or substitution procedures were required.

References:

1. Levine ME, et al. An epigenetic biomarker of aging for lifespan and healthspan. Aging (Albany NY) 10, 573-591 (2018).
2. Lu AT, et al. DNA methylation GrimAge strongly predicts lifespan and healthspan. Aging (Albany NY) 11, 303-327 (2019).
3. Belsky DW, Caspi A, Corcoran DL, Sugden K, Poulton R, Arseneault L, Baccarelli A, Chamarti K, Gao X, Hannon E, Harrington HL, Houts R, Kothari M, Kwon D, Mill J, Schwartz J, Vokonas P, Wang C, Williams BS, Moffitt TE. DunedinPACE, a DNA methylation biomarker of the pace of aging. Elife. 2022 Jan 14;11:e73420.

Line 403 – Prevalent cardiometabolic disease may be another confounder of dietary pattern and epigenetic age. Individuals with prevalent disease may be following specific dietary patterns because of this. Prevalent cardiometabolic disease (diabetes, hypertension) is associated with accelerated aging. You account for BMI, but there are metabolically healthy individuals who are overweight, and metabolically unhealthy individuals at normal BMIs. Your effect estimates were slightly attenuated when including BMI in the model, therefore may be worth considering cardiometabolic diseases.

We agree that prevalent cardiometabolic disease could potentially confound the associations between diet quality and epigenetic aging if individuals with disease have altered their dietary patterns as a consequence of diagnosis.[1-4] However, we would like to note that cardiometabolic factors such as diabetes and hypertension may also lie on the causal pathway between diet quality and epigenetic aging, rather than acting solely as confounders. Indeed, the slight attenuation of effect estimates observed after adjustment for BMI (Supplementary Figure 3; Supplementary Table 3) is consistent with BMI possibly being a partial mediator of the diet-epigenetic aging relationship.[5,6] In such cases, adjustment for these factors could block part of the causal effect of interest and potentially introduce bias.

To address the reviewer's concern while acknowledging this complexity, we conducted additional sensitivity analyses excluding participants with prevalent hypertension (n = 2,453 excluded; analytic sample n = 4,017) and diabetes at baseline (n = 436 excluded; analytic sample n = 6,034). Associations remained largely consistent in direction and magnitude across both sensitivity analyses (Supplementary Table 3). Some associations with PhenoAA were attenuated when excluding participants with hypertension (e.g., Nordic, DASH), which may reflect the substantial reduction in sample size (38%) and/or the role of blood pressure-related pathways in mediating the diet-PhenoAA relationship. Importantly, when excluding only participants with diabetes (7% sample reduction), results were highly consistent with the main findings (Model 1). Together, these sensitivity analyses suggest that while cardiometabolic conditions may partially mediate the diet-epigenetic aging relationship, the core findings are robust and not driven by confounding due to disease-related dietary changes.

Changes in manuscript and supplementary files:

- Methods section updated to describe how the prevalence of hypertension and diabetes was defined (page 18, lines 492-498), and additional sensitivity analyses (page 18, lines 521): "*To address potential confounding by prevalent cardiometabolic disease, we repeated the main analyses after excluding participants with hypertension or diabetes.*"
- Supplementary Table S3 updated to include Model 4 (sensitivity analysis excluding participants with prevalent hypertension), and Model 5 (sensitivity analysis excluding participants with prevalent diabetes).
- Results section updated to note the sensitivity analysis excluding participants with prevalent hypertension and diabetes (page 7, lines 167-170) *"When excluding participants with prevalent hypertension (Model 4; n = 4,017) or diabetes (Model 5; n = 6,034), the associations remained*

*consistent in direction and magnitude with the main findings, suggesting that prevalent cardiometabolic disease does not substantially confound our results (**Suppl. Table 3**).”*
• Discussion (pages 12, lines 318-324): *“Sensitivity analyses excluding participants with prevalent hypertension or diabetes yielded associations consistent in direction and magnitude with the main findings, suggesting that the observed relationships are not driven by confounding due to disease-related dietary changes. The partial attenuation of some associations in these analyses, as well as after adjustment for BMI, is consistent with cardiometabolic factors potentially lying on the causal pathway between diet quality and epigenetic aging rather than acting solely as confounders.”*

References:
1. Xiang X. Chronic disease diagnosis as a teachable moment for health behavior changes among middle-aged and older adults. J Aging Health. 2016;28(6):995-1015.
2. Velentzis LS, et al. Significant changes in dietary intake and supplement use after breast cancer diagnosis in a UK multicentre study. Breast Cancer Res Treat. 2011;128(2):473-82.
3. Cha E, Choi Y, Bancks M, Faulkner MS, Dunbar SB, Umpierrez GE, et al. Longitudinal changes in diet quality and food intake before and after diabetes awareness in American adults: the Coronary Artery Risk Development in Young Adults (CARDIA) study. BMJ Open Diabetes Research & Care. 2024;12:e003800.
4. Rigby RR, Williams LT, Mitchell LJ, Ball L, Hamilton K. Understanding dietary behaviour change after a diagnosis of diabetes: A qualitative investigation of adults with type 2 diabetes. PLoS One. 2022 Dec 12;17(12):e0278984.
5. Li DL, Hodge AM, Cribb L, Southey MC, Giles GG, Milne RL, Dugué PA. Body Size, Diet Quality, and Epigenetic Aging: Cross-Sectional and Longitudinal Analyses. J Gerontol A Biol Sci Med Sci. 2024 Apr 1;79(4):glae026.
6. Franzago M, Pilenzi L, Di Rado S, Vitacolonna E, Stuppia L. The epigenetic aging, obesity, and lifestyle. Front Cell Dev Biol. 2022 Sep 13;10:985274.

Line 421 – Why did you select these clocks vs. first and third generation?

We focused on second-generation epigenetic clocks (GrimAge and PhenoAge) because these clocks were explicitly developed to capture biological aging processes rather than chronological age. First-generation clocks (e.g., HorvathAge, HannumAge) were trained primarily to predict chronological age from DNA methylation patterns. While accurate for age prediction, they have been shown to be less sensitive to lifestyle and environmental exposures and weaker predictors of health outcomes compared to later-generation clocks.[1-3]

Later-generation clocks integrate additional physiological and clinical information. Both our previous work and independent studies have demonstrated that these clocks better reflect inter-individual variability in biological aging, multi-system dysfunction, and mortality risk compared with first-generation clocks.[4-5]

Following the reviewer’s suggestion, we have additionally included a third-generation epigenetic aging marker, DunedinPACE, which was designed to measure the pace of biological aging.[6] The inclusion of DunedinPACE allowed us to complement the second-generation clocks by capturing a dynamic aspect of aging. We found that across all ten diet quality scores, associations with DunedinPACE were statistically significant after Benjamini-Hochberg FDR correction, with healthier dietary patterns associated with lower DunedinPACE (including the EAT-Lancet diet), and less healthy patterns (uPDI and DII) associated with higher DunedinPACE, reinforcing our main findings. Notably, effect sizes were consistently larger for DunedinPACE than for GrimAA and PhenoAA. We believe this difference reflects fundamental distinctions in what these clocks measure. GrimAge and PhenoAge were trained on cross-sectional data to estimate cumulative biological age, essentially representing the total burden of biological

aging that has accumulated over the lifespan. In contrast, DunedinPACE was developed using longitudinal data from the Dunedin Study, tracking within-person changes across 19 biomarkers over time to capture the current rate of biological aging rather than the accumulated state. Diet, as a modifiable and dynamic exposure, may more readily influence the ongoing pace of aging than reverse decades of accumulated biological wear. This interpretation aligns with our observation that the EAT-Lancet diet showed little to no association with GrimAA and PhenoAA but was significantly associated with DunedinPACE, suggesting that this dietary pattern may influence the pace of biological aging despite not showing associations with markers of accumulated biological age. The direction and overall pattern of associations nonetheless remained consistent across all three epigenetic aging outcomes. .

We have updated Figure 2, Supplementary Table 2-6, and the results section correspondingly (page 6, line 139-159).

We have also updated the Methods section to explicitly justify our focus on second- and third-generation clocks (page 17, lines 461-465): *"Both our previous work and studies by others have shown that second- and third-generation epigenetic clocks better capture inter-individual variability in biological aging processes and multi-system dysfunction than first-generation clocks, which primarily reflect chronological age. Therefore, we focused our analyses on second- and third-generation measures, including PhenoAge, GrimAge, and DunedinPACE."*

References:

1. McCrory C et al. GrimAge Outperforms Other Epigenetic Clocks in the Prediction of Age-Related Clinical Phenotypes and All-Cause Mortality. J Gerontol A Biol Sci Med Sci. 2021;76(5):741-749. PMID: 33211845
2. Levine ME et al. An epigenetic biomarker of aging for lifespan and healthspan. Aging (Albany NY). 2018;10(4):573-591. PMID: 29676998
3. Lu AT, et al. DNA methylation GrimAge strongly predicts lifespan and healthspan. Aging (Albany NY) 11, 303-327 (2019).
4. Liu D, Aziz NA, Landstra EN, Breteler MMB. The lipidomic correlates of epigenetic aging across the adult lifespan: A population-based study. Aging Cell. 2023 Sep;22(9):e13934. doi: 10.1111/acel.13934. Epub 2023 Jul 26.
5. Liu D, Aziz NA, Pehlivan G, Breteler MMB. Cardiovascular correlates of epigenetic aging across the adult lifespan: a population-based study. Geroscience. 2023 Jun;45(3):1605-1618. doi: 10.1007/s11357-022-00714-0. Epub 2023 Feb 8.
6. Belsky DW, Caspi A, Corcoran DL, Sugden K, Poulton R, Arseneault L, Baccarelli A, Chamarti K, Gao X, Hannon E, Harrington HL, Houts R, Kothari M, Kwon D, Mill J, Schwartz J, Vokonas P, Wang C, Williams BS, Moffitt TE. DunedinPACE, a DNA methylation biomarker of the pace of aging. Elife. 2022 Jan 14;11:e73420.

Line 424 – Please explain more about the adjustment for batch effects that was performed. Why not chip? Row?

We adjusted for batch effects by including chip, row, and the first five control probe principal components in all analyses. Chip and row were included to account for technical variation related to array processing and sample positioning, while control probe principal components capture additional unmeasured technical variation arising from laboratory processing and array performance. We have now clarified this explicitly in the Methods section.

Page 18, line 511-516: *"To investigate the relationship between dietary quality and epigenetic age acceleration metrics, we used multivariable linear regression models. Models were adjusted for sex,*

smoking status, total energy intake, batch effects (including chip, row and first five control probe principal components) and blood cell proportion estimated from the same DNA methylation samples."
Page 19, line 545-549: "*We investigated the relationship between diet quality scores (independent variables) and DNA methylation level (dependent variable) across the epigenome using multiple linear regression, adjusting for age, sex, batch effects (including chip, row and first five control probe principal components), blood cell proportion, the first ten genetic principal components to account for population stratification, smoking status and total energy intake (Model 1)."*

Line 460 – Specify EPIC v1.0 now that there are multiple versions.

We have now specified that the EPIC v1.0 was used in our study throughout the Methods section in the main manuscript (page 16, line 457; page 20, line 563), and in the Supplementary methods.

Results

Line 168 – Consider including the age range for EPIC – Potsdam in parentheses as you did for Rhineland.

Thank you for this suggestion. We have now included the age range for EPIC-Potsdam in the Results section for consistency with the Rhineland Study description (page 7, line 191): *"The mean age of the participants was 49.9 years (SD = 8.9), with 62% being women. The age range was 35–64 years for women and 40–64 years for men."*

Line 168 – Why only 1k people from EPIC Potsdam? Was this not a much larger study? Who was included?

The EPIC-Potsdam study is indeed a large population-based cohort with approximately 27,000 participants.[1] DNA methylation was measured using the Illumina MethylationEPIC v1.0 array in a randomly selected subcohort of EPIC-Potsdam (n= 1147) as part of a nested case-cohort design.[2] The 1,034 participants included in our replication analysis represent those with available EPIC v1.0 array methylation data and complete dietary and covariate information at the time of our analysis. This subsample is broadly representative of the overall EPIC-Potsdam cohort in terms of key demographic characteristics. The sample size is consistent with other recent methylation studies from EPIC-Potsdam[2] and provides adequate statistical power for replication of epigenome-wide findings, as demonstrated by our successful validation of associations for diet quality scores with epigenetic aging markers and CpG sites.

References:
1. Boeing H, et al. Recruitment procedures of EPIC-Germany. Ann Nutr Metab. 1999;43(4):205-15.
2. Eroglu, B., Eichelmann, F., Kuxhaus, O. et al. Trace element-linked DNA methylation sites and their association with type 2 diabetes and cardiovascular diseases: EPIC-Potsdam cohort study. Clin Epigenet 17, 172 (2025).

Line 183 – "significant differences" are described but no test statistics are presented. These results should be reported on the figure (Supp Fig 8) or in a supplementary table.

We have now provided the corresponding test statistics for the replication cohort analyses in Supplementary Tables 10 and 11, including full regression outputs (β, SE, test statistics, 95% CI, nominal p-values, and BH-FDR q-values) for EPIC-Potsdam for the main model (Model 1), the BMI-adjusted

sensitivity model (Model 2), and the smoking-stratified analyses. These additions support the subgroup comparisons referenced in the text and Supplementary Figure 9 (formerly Supplementary Figure 8).

In addition, we have added a meta-analysis results table reporting pooled inverse-variance weighted estimates with BH-FDR q-values (Supplementary Table 12). We have also revised the Results text to reference these tables and to clearly distinguish nominal from BH-FDR-significant findings (page 7-8, lines 348-364).

Lines 244-249: it is mentioned that several CpGs associated with mQTLs, but no information is provided about these in the text or a supplemental table, which makes this statement hard to interpret. For CpGs that are present on the 450K array, it would be possible to perform an enrichment test of whether more mQTL-associated CpGs are detected than expected by chance. (A similar test could also be helpful to help interpret the eQTM results.)

Information on mQTLs was provided in Supplementary Table 8, but this was not explicitly referenced in the original text. We have now clarified this in the manuscript (page 10, line 266).

Following the reviewer's suggestion, we additionally performed enrichment analyses to assess whether EWAS-identified CpGs for each diet quality score were over-represented among CpGs with known mQTLs. This analysis aimed to evaluate whether the identified CpGs are more likely to be genetically regulated than expected by chance. Enrichment was tested using Fisher's exact tests, restricting both EWAS-identified CpGs and background CpGs to those present on the Illumina 450K array and tested in our EWAS (conducted on the 850K array), as currently available mQTL databases are primarily based on the 450K platform. We found that none of the dietary trait-associated CpGs showed significant enrichment for mQTLs after correction for multiple testing (all FDR-adjusted p-values > 0.05; **Supplementary Table 21**). These results suggest that the identified CpGs overlapping the 450K array are not predominantly driven by known genetic regulation. As mQTL resources for the EPIC/850K array become available in the future, similar analyses could be extended to CpGs unique to the 850K platform.

We have added the following text to the Methods (Page 21, line 607-612): *"To evaluate whether the identified CpGs are more likely to be genetically regulated than expected by chance, we additionally performed enrichment analyses using Fisher's exact tests, restricting both EWAS-identified CpGs and background CpGs to those present on the Illumina 450K array and tested in our EWAS (conducted on the 850K array), as currently available mQTL databases are primarily based on the 450K platform."*

Added Results section (Page 10, line 275-280): "*We found that none of the dietary trait-associated CpGs showed significant enrichment for mQTLs after correction for multiple testing (all FDR-adjusted p-values > 0.05;* ***Suppl. Table 21****). These results suggest that the identified CpGs overlapping the 450K array are not predominantly driven by known genetic regulation. As mQTL resources for the EPIC/850K array become available in the future, similar analyses could be extended to CpGs unique to the 850K platform.*"

Regarding the eQTM analyses, in addition to the look-up in the BIOS database (Results shown in **Suppl. Table 4**), we extended our investigation using individual-level gene expression data from the Rhineland Study. This approach allowed us to directly assess associations between the identified CpGs and the expression levels of their mapped genes, rather than relying on overlap-based enrichment analyses. Using this individual-level data, we found that 33 out of 93 diet-related CpGs were significantly associated with gene expression (Figure 5), providing direct evidence of functional relevance. Given that these results are

based on direct association testing using individual-level data, we did not perform an additional enrichment analysis for eQTMs.

As mentioned above, 20 tests are performed in Figure 2, with many reported as “significant”, but no multiple testing appears to be applied.

We have addressed this by applying multiple-testing correction to the analyses presented in Figure 2. Specifically, we now control the false discovery rate using the Benjamini–Hochberg (BH) procedure for all associations between diet quality scores and epigenetic age acceleration (i.e., across the full set of diet score × clock tests shown in Figure 2). We additionally provide complete regression outputs in the Supplementary Tables for the main Figure 2 analyses and all sensitivity and stratified models, including β, SE, test statistics, 95% CIs, nominal p-values, and BH-FDR q-values (and interaction p-values where applicable). Finally, all forest plots of diet–epigenetic age acceleration associations explicitly indicate BH-FDR significance via point shapes corresponding to BH-FDR categories. Thus, while nominal p-values are shown, statistical significance is now interpreted based on BH-FDR–adjusted q-values.

Discussion

Line 254 – How many people were smokers and adhered to any of the healthy dietary patterns?

Because diet quality scores are continuous and do not have universal adherence cut-offs, we addressed this by summarizing diet score distributions by smoking status using pre-defined quartiles. We now report the number of participants in each smoking category and the proportion falling in the healthiest quartile for each diet score in Supplementary Table 20. Overall, current smokers (n = 767) were consistently less likely to fall in the healthiest quartile compared with former (n = 2,653) and never smokers (n = 3,050). For example, 13.6% of current smokers were in the healthiest AHEI quartile versus 25.4% of former and 26.5% of never smokers; similarly for DASH (13.0% vs 22.7% vs 24.7%) and the Nordic diet score (16.9% vs 24.7% vs 26.1%). This pattern was observed across most diet scores and provides context for the smoking-stratified analyses, indicating that the stronger associations observed among current smokers are unlikely to be driven by a higher overall distribution of healthy diet scores in this subgroup.
We have added this clarification to the Discussion (page 11, lines 314-318): *“The stronger association between diet quality scores with epigenetic aging acceleration in current smokers, which was also replicated in the EPIC-Potsdam, was observed despite current smokers being less likely to fall in the healthiest quartile across most diet scores (Supplementary Table 20), suggesting that this pattern is unlikely to be explained by higher overall diet quality among smokers.”*

Line 282 – Here you discuss the similarities in findings between the training and validation cohorts, but how do you make sense of the differences in what you found between the two?

Thank you for raising this point. We would like to clarify that the findings between the discovery and replication cohorts were largely consistent rather than discrepant. Across the epigenetic aging analyses, the direction of associations was concordant for nearly all diet quality scores (Supplementary Figures 7 & 8). The meta-analysis confirmed these patterns, reinforcing the robustness of our findings.

For the EWAS results, while not all CpGs reached nominal significance in the replication cohort, the overall concordance was strong: more than 50% of top CpGs retained consistent effect directions even when not reaching statistical significance (Supplementary Figures 9-12). This pattern is expected in

replication studies, particularly given the smaller sample size of EPIC-Potsdam (n = 1,034 vs. n = 6,470 in the Rhineland Study) and the temporal differences in data collection.

As we note in the Discussion (page 14, lines 405-411), some variance in effect estimates may be attributable to differences in when dietary data were collected (1990s in EPIC-Potsdam vs. more recently in the Rhineland Study), potentially reflecting different eating patterns, as well as possible regional variations. Despite these nuances, the overall concordance in both direction and magnitude of associations supports the generalizability of our findings.

Line 333 – Based on inclusion criteria, specify German population of Caucasian ancestry. What is the ancestry of the EPIC-Potsdam cohort?

Like the Rhineland Study, participants in the EPIC-Potsdam study predominantly have German/European ancestry. We have revised the Method and Discussion to specify the ancestry of both cohorts. We have updated the text accordingly:
Discussion, page 14, line 397-399: *"Our findings are based on a predominantly German population of European ancestry in both cohorts, and generalizability to persons with other ancestries or dietary habits remains to be investigated."*
Methods, Page 16, line 431-432: *"The Rhineland Study participants are predominantly German of Caucasian descent."*
Methods, Page 19, line 556: *"Like the Rhineland Study, the participants of EPIC-Potsdam were predominantly German of Caucasian ancestry."*

Tables & Figures
Table 1 - Here and in other relevant descriptive tables:
- Consider including a descriptor of socioeconomic status in these tables as this is another potential confounder.

In Table 1 and Table S1, we clarified that education level (coded according to the International Standard Classification of Education; ISCED 2011) is presented as a proxy indicator of socioeconomic status.

- Include a footnote that describes how high/medium/low education are classified

We have now added a footnote defining the education categories: low (lower secondary education or below), middle (upper secondary education to undergraduate university level), and high (postgraduate university study).

- Consider including prevalence of other cardiometabolic diseases aside from obesity (i.e. diabetes, hypertension)

We have now included the prevalence of additional cardiometabolic conditions, hypertension and diabetes, in the population characteristics tables (Table 1 and Table S1).

- Are you reporting the mean residual value for GrimAA and PhenoAA? What were the actual GrimAge and PhenoAge values?

Yes, we report the residual values for GrimAA and PhenoAA in Table 1 and Supplementary Table 1. These residuals represent epigenetic age acceleration, defined as the difference between epigenetic age and chronological age after regressing epigenetic age on chronological age. This approach is standard in the epigenetic aging literature[1,2] and directly captures the phenotype of interest, whether an individual is biologically older or younger than expected for their chronological age. Reporting the raw GrimAge and PhenoAge values would be less informative, as these are highly correlated with chronological age by design. We have added a footnote to Table 1 and Supplementary Table 1 to clarify that GrimAA and PhenoAA represent age acceleration residuals.

References:
1. Levine ME et al. An epigenetic biomarker of aging for lifespan and healthspan. Aging (Albany NY). 2018;10(4):573-591. PMID: 29676998
2. Lu AT et al. DNA methylation GrimAge strongly predicts lifespan and healthspan. Aging (Albany NY). 2019;11(2):303-327. PMID: 30669119

Supplementary Tables 11 and 12 lack important information such as citations to the previous studies indicated, as well as the cg names involved.

The CpGs corresponding to previously reported EWAS and GWAS traits are provided in Supplementary Table 8, which explicitly lists the CpG-trait pairs. This table is intended to provide detailed, CpG-specific information linking the identified diet-associated CpGs to previously reported EWAS/GWAS findings.

Supplementary Tables 11 and 12 (now updated to Supplementary Tables 18 and 19) serve a different purpose. Rather than repeating CpG-level details, these tables summarize the overlap of previously reported EWAS/GWAS traits across the different diet quality scores, providing a trait-centered perspective that facilitates comparison across dietary patterns. For clarity, CpG-level information and literature citations are therefore not repeated in these summary tables but are instead referenced back to Supplementary Table 8. As shown in Supplementary Tables 18 and 19, the CpGs and genes associated with the identified dietary patterns have previously been linked to a broad range of traits, including diet-related behaviors (e.g., alcohol consumption and smoking), cardiometabolic traits (e.g., type 2 diabetes, BMI, waist circumference, CRP, insulin and glucose levels, and blood pressure), lipid traits (e.g., HDL, LDL, and triglycerides), circulating protein levels, and mortality.

In addition to Supplementary Figure 7, scatterplots like Supplementary Figure 11 (but with just one point per measure) could be informative to show the correspondence between the effect sizes across discovery (Figure 2) and replication (Supp Fig 7) for the different measures.

We have added Supplementary Figure 8, which presents scatterplots comparing discovery (Rhineland Study) and replication (EPIC-Potsdam) β estimates for the associations between diet quality scores and epigenetic age acceleration. Each point represents one diet quality score, with separate panels for GrimAA and PhenoAA and for Models 1 and 2. The cross-cohort concordance was high, with Pearson correlations ranging from r = 0.79–0.83 for GrimAA and r = 0.83–0.91 for PhenoAA.
We have updated the Results section accordingly (page 8, lines 351-353):
"This cross-cohort correspondence was further supported by high concordance of effect estimates across diet scores (Suppl. Figure 8), with Pearson correlations ranging from r = 0.79–0.83 for GrimAA and r = 0.83–0.91 for PhenoAA across Models."

Supplementary Figure 11 – Correlation should be reported separately by measure; reporting correlation of effect sizes for all measures combined is likely to be misleading, especially given the differences in scale seen in the figure.

We agree that reporting correlations separately by diet quality score is informative. As described in the Results section (page 8, lines 214-220), we report both the overall cross-cohort correlation and the diet-specific correlations:
"Beta-beta correlation analyses of the epigenome-wide significant CpGs (identified in the discovery cohort) further supported the consistency of the findings between the Rhineland Study and EPIC-Potsdam (r = 0.54, p < 0.001) (Suppl. Figure 12 - formerly 11). Stratified analyses by diet quality score revealed varying degrees of correlation, with the highest reproducibility observed for CpGs associated with AHEI (r = 0.85), uPDI (r = 0.92), and DII (r = 0.67), while lower correlations were seen for CpGs linked to Nordic (r = 0.09) and MIND (r = 0.25) diets (Suppl. Figure 13)."
We believe presenting both the aggregate and stratified correlations provides a comprehensive picture of replication consistency across dietary patterns.

Supplementary Table 10 – "Pathways" is misspelled. Please specify what columns DE and P.DE stand for.

We corrected the typographical error in "Pathways" and added definitions for the DE and P.DE columns in the table footnote (DE: number of mapped diet-associated genes within the pathway; P.DE: enrichment p-value for over-representation).

Supplementary Figures 15 and 16 – What does FDR refer to in the legend? Assuming FDR correction. Please describe in footnote.

We revised Figures S15 and S16 legends to define FDR explicitly as the false discovery rate (Benjamini–Hochberg adjusted p-values) used to correct enrichment results for multiple testing.

Reviewer #3 (Remarks to the Author):

I co-reviewed this manuscript with one of the reviewers who provided the listed reports. This is part of the Nature Communications initiative to facilitate training in peer review and to provide appropriate recognition for Early Career Researchers who co-review manuscripts.

Thank you very much for your time and effort.

Reviewer #4 (Remarks to the Author):

The authors performed a series of elegant analyses examining the associations of 10 different diet quality scores with markers of accelerated epigenetic aging and DNA methylation profiles. The discovery sample was derived from the Rhineland Study, a

population-based study of individuals aged 30 years or older. The authors sought replication of their findings in participants from the EPIC-Potsdam study.
The study reports associations between higher "healthy" dietary pattern adherence scores (except for the EAT-Lancet score) and lower epigenetics indices of biological age, and between higher "unhealthy" dietary pattern adherence scores and higher epigenetic indices of biological age. Stratified analyses revealed that these associations were driven (or were generally stronger) amongst current smokers compared to former and never smokers. In EWAS analyses, except for the EAT-Lancet score, diet scores exhibited epigenome-wide significant associations with DNA methylation sites, with mostly negative beta coefficients for 'healthy" dietary patterns and positive beta coefficients for "unhealthy" dietary patterns.
This work provides valuable insights into the potential influences of diet on biological aging. Nevertheless, there are certain methodological issues that warrant further attention; these are outlined below.

The authors should consider adjusting the associations between diet quality scores and markers of accelerated epigenetic aging for physical activity (at least as a sensitivity analysis). It is possible that people with higher diet quality scores also maintain an overall healthier lifestyle and are engaged in regular physical exercise. Physical activity has been associated with slower epigenetic aging, therefore, potentially acting as a confounder in the reported associations.

We appreciate the reviewer's suggestion to consider physical activity as a potential confounder. Indeed, physical activity has been associated with slower epigenetic aging, including in the Rhineland Study population, where Fox et al.[1] reported inverse associations between objectively measured physical activity and epigenetic age acceleration.

In the revised manuscript, we conducted an additional sensitivity analysis adjusting for objectively measured physical activity (MET-hours/day, derived from accelerometry; Table S3, Model 3). Consistent with the BMI-adjusted model, inclusion of physical activity attenuated the associations between diet quality scores and epigenetic age acceleration metrics. However, an important caveat is that accelerometer-derived physical activity data were available for only approximately half of the participants in our analytical sample (n = 3,209), limiting statistical power and generalizability of this sensitivity analysis. To disentangle whether the attenuated associations were due to reduced sample size or confounding by physical activity, we additionally ran the base model (Model 1) restricted to the subset of participants with accelerometer data but without adjusting for physical activity. The estimates from this restricted sample were very similar to those from the physical activity-adjusted model, suggesting that the attenuation is primarily attributable to reduced statistical power rather than confounding by physical activity. We therefore present this as a supplementary sensitivity model rather than a primary analysis.

<u>Changes in manuscript and supplementary files:</u>

- Methods section updated to describe physical activity assessment (page 17, lines 697-715): "*Physical activity was recorded with accelerometers and reported as average daily energy expenditure in metabolic equivalents (METs) per hour*", and additional sensitivity analyses (page 18, lines 742-748): "*To evaluate the effect of adiposity and physical activity, we additionally adjusted for BMI and MET/hours in sensitivity analysis*".
- Supplementary Table S3 updated to include Model 3 (adjusted for physical activity).
- Results section updated to note the physical activity-adjusted sensitivity analysis (page 7, lines 314-317): "*Similarly, further adjustment for objectively measured physical activity (n = 3,209*

*participants with accelerometer data) attenuated these associations, which appears attributable to the reduced sample size rather than confounding, as similar attenuation was observed when restricting the sample without adjustment for physical activity. (**Suppl. Table 3, Model 3**).”*

References:
1. Fox FAU, Liu D, Breteler MMB, Aziz NA. Physical activity is associated with slower epigenetic ageing-Findings from the Rhineland study. Aging Cell. 2023 Jun;22(6):e13828.

The authors should consider presenting the beta coefficients and the respective confidence intervals of the reported associations between higher diet quality scores and epigenetic aging indices in a table format or in the narrative. It is rather difficult for the reader to determine from Figure 2 which associations are significant since, for some associations such as those of the MDS, MIND, and PDI with GrimAA it appears as the confidence interval band intercepts the null (horizontal) line.

We appreciate the reviewer's suggestion to improve the clarity of the associations between diet quality scores and epigenetic aging indices. This concern aligns with feedback from another reviewer regarding the presentation of test statistics and multiple-testing adjustment. In the revised manuscript, we therefore strengthened the presentation across all diet-epigenetic clock analyses by (i) providing complete regression outputs in supplementary tables (β, SE, test statistics, 95% CIs, nominal p-values, and BH-FDR q-values), and (ii) updating all relevant forest plots so statistical significance is explicitly indicated via point shapes corresponding to BH-FDR significance categories. This facilitates interpretation of Figure 2 and related sensitivity and stratified analyses.

<u>Changes in manuscript and supplementary files:</u>

• Figure 2 updated for clearer visualization of statistical significance (point shapes correspond to BH-FDR significance categories).
• Supplementary Figures S3–S5 updated so point shapes reflect BH-FDR significance categories (BMI-adjusted sensitivity model and stratified analyses).
• Supplementary Table S3 provides full regression results underlying Figure 2a (Model 1; also includes the BMI-adjusted sensitivity Model 2).
• Supplementary Table S6 provides full regression results underlying Figure 2b (smoking-stratified analysis), including interaction p-values and BH-FDR-adjusted interaction q-values.
• Supplementary Tables S4-S5 provide full regression outputs for stratified analyses by sex (Table S4) and BMI category (Table S5), including interaction terms and multiple-testing adjustment (β, SE, test statistics, 95% CIs, nominal p-values, BH-FDR q-values, interaction p-values, and BH-FDR–adjusted interaction q-values).

It is somewhat unclear why in the replication cohort the nominal threshold for statistical significance ($p < 0.05$) was used for EWAS analyses instead of the epigenome-wide significance level ($p < 5.95 \times 10^{-8}$). A potentially more robust replication approach would include EWAS studies being conducted in parallel in the two independent cohorts (without using the findings in the discovery cohort to inform replication), epigenome-wide significant associations being determined independently, and then assessing the overlap of the identified associations between the two cohorts?

We thank the reviewer for raising this important methodological point. The replication strategy we employed, which is applying an epigenome-wide significance threshold in the discovery cohort followed by a nominal significance threshold ($p < 0.05$) with consistent effect direction in the replication cohort,

follows established practice in epigenome-wide association studies.[1-4] The rationale for this approach is that the stringent Bonferroni-corrected threshold ($p < 5.95 \times 10^{-8}$) applied in the discovery phase appropriately controls for multiple testing across 840,896 CpGs in our study. In the replication phase, we evaluated only the pre-specified set of CpGs that reached epigenome-wide significance in discovery, thereby substantially reducing the multiple testing burden. Requiring epigenome-wide significance independently in both cohorts would be overly conservative and likely underpowered to detect true associations, particularly given the smaller sample size of the EPIC-Potsdam replication cohort (n = 1,034) compared to the Rhineland Study discovery cohort (n = 5,768).

The reviewer's suggested approach, conducting parallel EWAS in both cohorts and assessing overlap, is a valid alternative strategy. However, this approach could potentially reduce statistical power in smaller cohorts and miss true associations that do not reach epigenome-wide significance in both samples independently. Our approach prioritizes replication of the most robust discovery signals while requiring both nominal statistical significance and directional consistency in the replication cohort.

We have clarified the rationale for our replication strategy in the Methods section (page 20, lines 576-584): *"To confirm our EWAS findings, we examined whether the epigenome-wide significant CpGs identified in the discovery cohort (p-value < 5.95×10^{-8}) were replicated (p-value < 0.05) in the EPIC-Potsdam cohort. CpGs were classified into three replication categories: "Yes" for CpGs reaching nominal significance (p < 0.05) with consistent effect direction; "Partially" for CpGs with consistent effect directions but p > 0.05; and "No" for CpGs without consistent effect directions. This discovery-replication framework, in which a stringent threshold is applied in discovery and nominal significance with directional consistency is required for replication, is an established approach in EWAS that balances type I error control with statistical power."*

References:

1. Ma J, et al. Whole Blood DNA Methylation Signatures of Diet Are Associated With Cardiovascular Disease Risk Factors and All-Cause Mortality. Circ Genom Precis Med. 2020
2. Do WL, et al. Epigenome-wide association study of diet quality in the Women's Health Initiative and TwinsUK cohort. Int J Epidemiol. 2021;50(2):675-684.
3. Mansell G, et al. Guidance for DNA methylation studies: statistical insights from the Illumina EPIC array. BMC Genomics. 2019;20:366.
4. Campagna MP, Xavier A, Lechner-Scott J, Maltby V, Scott RJ, Butzkueven H, Jokubaitis VG, Lea RA. Epigenome-wide association studies: current knowledge, strategies and recommendations. Clin Epigenetics. 2021 Dec 4;13(1):214.

It is somewhat unclear why the GrimAA and PhenoAA epigenetic age acceleration metrics were selected compared to other measures. For example, a recent study that tested whether the pace of biological aging mediates the relationships between adherence to a healthy diet and incident dementia and mortality, used the DunedinPACEepigenetic clock to measure the pace of biological aging (PMID: 38407506). This index has the advantage that it was derived from 19 biomarkers ascertained longitudinally, preventing short-term illnesses from affecting aging measurements, and capturing gradual, long-term changes that truly reflect aging. The authors should consider further justifying their selection of epigenetic metrics, describe potential advantages and pitfalls compared to other measures that led them to this selection, and outline potential differences in the aspects of biological aging each measure captures (i.e., how are these measures similar and how they differ).

We thank the reviewer for this valuable suggestion, which was also raised by Reviewer #2. As detailed in that response, we have now incorporated DunedinPACE into our analyses.

Briefly, we observed consistent associations between diet quality scores and DunedinPACE. All ten diet quality scores showed statistically significant associations with DunedinPACE after Benjamini-Hochberg FDR correction, with greater adherence to healthy dietary patterns (including the EAT-Lancet diet) associated with a slower pace of biological aging and higher adherence to unhealthy dietary patterns (uPDI and DII) associated with a faster pace of aging.

Notably, the effect sizes were consistently larger for DunedinPACE compared to GrimAA and PhenoAA, which likely reflects fundamental differences in what these metrics capture: GrimAge and PhenoAge estimate cumulative biological age from cross-sectional data, whereas DunedinPACE was developed using longitudinal within-person biomarker trajectories to measure the current rate of aging. Modifiable exposures such as diet may may more readily influence both the ongoing pace of biological aging than reverse accumulated biological burden. Because DunedinPACE represents a rate of aging rather than an age-acceleration residual, models with DunedinPACE as the outcome were additionally adjusted for chronological age.

We have updated Figure 2, Supplementary Tables 2–6, and the results section correspondingly (page 6, line 140-159), and added rationale for our clock selection[1-3] in the Methods section (page 17, line 461-465): *"Both our previous work and studies by others have shown that second- and third-generation epigenetic clocks better capture inter-individual variability in biological aging processes and multi-system dysfunction than first-generation clocks, which primarily reflect chronological age. Therefore, we focused our analyses on second- and third-generation measures, including PhenoAge, GrimAge, and DunedinPACE."*

References:
1. Levine ME et al. An epigenetic biomarker of aging for lifespan and healthspan. Aging (Albany NY). 2018;10(4):573-591. PMID: 29676998.
2. Lu AT et al. DNA methylation GrimAge strongly predicts lifespan and healthspan. Aging (Albany NY). 2019;11(2):303-327. PMID: 30669119.
3. Belsky DW, Caspi A, Corcoran DL, Sugden K, Poulton R, Arseneault L, Baccarelli A, Chamarti K, Gao X, Hannon E, Harrington HL, Houts R, Kothari M, Kwon D, Mill J, Schwartz J, Vokonas P, Wang C, Williams BS, Moffitt TE. DunedinPACE, a DNA methylation biomarker of the pace of aging. Elife. 2022 Jan 14;11:e73420.

The authors evaluated potential effect modification of the diet quality scores by sex, smoking status, and BMI on epigenetic age acceleration. What were the generating hypotheses behind these analyses? The authors should consider elaborating on that a bit more.

We thank the reviewer for this suggestion. The stratified analyses were motivated by prior evidence that smoking, BMI, and sex may modify epigenetic processes and/or diet-health associations.
Smoking is one of the most robust environmental exposures affecting DNA methylation patterns and has been consistently associated with accelerated epigenetic aging.[1,2] We hypothesized that the relationship between diet quality and epigenetic aging might differ by smoking status, potentially reflecting effect modification or differing baseline methylation states.

BMI has similarly been linked to altered DNA methylation profiles and epigenetic age acceleration, including our previous work.[3-5] Given that adiposity and diet are interrelated, we sought to assess whether associations were consistent across BMI categories or potentially modified by metabolic status.
For sex, we aimed to explore potential sex differences in the diet-epigenetic aging relationship, consistent with evidence of sex-specific patterns in epigenetic aging.[6,7] Stratification by sex is also standard epidemiological practice to explore sex differences.

We have added a brief statement to the Methods section clarifying the rationale for these stratified analyses (page 19, lines 530-533): *"We also evaluated effect modification of the diet quality scores by sex, smoking status, and BMI, as these factors have been associated with altered DNA methylation patterns and epigenetic age acceleration in prior studies."*

References:

1. Gao X, Zhang Y, Breitling LP, Brenner H. Relationship of tobacco smoking and smoking-related DNA methylation with epigenetic age acceleration. Oncotarget. 2016 Jul 26;7(30):46878-46889.
2. Lei MK, Gibbons FX, Simons RL, Philibert RA, Beach SRH. The Effect of Tobacco Smoking Differs across Indices of DNA Methylation-Based Aging in an African American Sample: DNA Methylation-Based Indices of Smoking Capture These Effects. Genes (Basel). 2020 Mar 14;11(3):311.
3. Horvath S, et al. Obesity accelerates epigenetic aging of human liver. Proc Natl Acad Sci USA. 2014;111(43):15538-15543.
4. Foster CA, et al. Epigenetic age acceleration correlates with BMI in young adults. Aging (Albany NY). 2023;15(2):513-523.
5. Liu D, Aziz NA, Pehlivan G, Breteler MMB. Cardiovascular correlates of epigenetic aging across the adult lifespan: a population-based study. Geroscience. 2023 Jun;45(3):1605-1618.
6. Kankaanpää A, et al. Do Epigenetic Clocks Provide Explanations for Sex Differences in Life Span? A Cross-Sectional Twin Study. J Gerontol A Biol Sci Med Sci. 2022;77(9):1898-1906.
7. Yusipov I, et al. Age-related DNA methylation changes are sex-specific: a comprehensive assessment. Aging (Albany NY). 2020;12(24):24057-24080.

In Supplemental Table 6 where the authors describe the results of the replication analysis of the EWAS study, it is somewhat unclear what "Partial" under the "Replicated" column means.

Specifically, CpGs were classified into three replication categories: "Yes" for CpGs that reached nominal significance ($p < 0.05$) in the replication cohort with consistent effect direction; "Partially" for CpGs with consistent effect directions but $p > 0.05$ in the replication cohort; and "No" for CpGs that did not show consistent effect directions.
We have now added a clear definition of the replication classification criteria both in the Methods section and as a footnote to Supplementary Table 6.

A potential limitation the authors should consider pointing out pertains to the generalizability of their findings in different race/ethnicities. There is evidence to suggest that the performance of DNA methylation-based predictors of chronological age (i.e., epigenetic clocks) is affected by race and ethnicity (PMID: 40205465). Therefore, the generalizability and external validity of the present findings to other racial/ethnic groups cannot be ascertained within the context of this study and needs to be further assessed.

We agree that evidence suggests DNA methylation-based predictors of biological age may perform differently across racial and ethnic groups, which has implications for the generalizability and external validity of our findings. We note that this limitation is acknowledged in the Discussion (page 14, lines 397-399), where we state: *'Our findings are based on a predominantly German population of European ancestry in both cohorts, therefore generalizability to persons with other ancestries or dietary habits remains to be investigated. '*
This statement encompasses concerns about both dietary pattern differences and potential variation in epigenetic marker performance across populations. We agree that future studies in more diverse populations are needed to confirm whether the observed associations between diet quality and epigenetic aging extend beyond European-ancestry groups.

In gene expression analyses, only 33 diet-associated CpGs were associated with their mapped gene expression levels. The authors should consider providing some tentative explanations on how identified associations between diet and the remaining DNA methylation sites (i.e. those not being associated with mapped gene expression levels), might be biologically relevant

We thank the reviewer for this insightful comment. There are several biologically plausible explanations for why the remaining diet-associated CpGs (i.e., those not associated with mapped gene expression levels) may still be biologically relevant:

First, tissue-specific methylation-expression relationships: our study measured both DNA methylation and gene expression in whole blood. However, dietary patterns may exert their primary molecular effects in metabolically relevant tissues such as adipose tissue, liver, or the gastrointestinal tract. Blood-based methylation changes may serve as biomarkers reflecting systemic dietary exposures, even if the functional transcriptional consequences occur in other tissues.[1,2]

Second, genomic context effects: many diet-associated CpGs are located in distal enhancers, gene body regions, or intergenic sequences where methylation-expression relationships are more complex. Enhancer methylation may regulate distal genes through long-range chromatin interactions, and the nearest annotated gene (used for standard mapping) may not be the actual regulatory target. Gene body methylation, in particular, is often positively (rather than inversely) correlated with transcription and may primarily affect alternative splicing or transcript isoform usage rather than total gene expression levels.[3,4]

Third, functions beyond transcriptional regulation: DNA methylation serves multiple biological functions independent of direct gene expression control, including maintenance of genome stability through silencing of repetitive elements (LINE-1, Alu sequences), regulation of chromatin structure and accessibility, and modulation of alternative splicing.[5,6]

Fourth, technical limitations: gene expression data were available for 93 of the 126 mapped genes, limiting our ability to detect all potential methylation-expression associations. Additionally, the effect sizes of methylation on expression are often subtle and may require larger sample sizes or different analytical approaches (e.g., cell-type-specific analyses) to detect reliably.

We have now added in the discussion (page 12-13, lines 347-359) a tentative explanation of the potential biological relevance of diet-associated CpGs that were not associated with mapped gene expression levels in our blood-based eQTM analysis.

References:

1. Braun PR, et al. Genome-wide DNA methylation comparison between live human brain and peripheral tissues within individuals. Transl Psychiatry 2019;9:47.
2. Edgar RD, et al. BECon: a tool for interpreting DNA methylation findings from blood in the context of brain. Transl Psychiatry 2017;7:e1187.Horvath S, et al. Obesity accelerates epigenetic aging of human liver. Proc Natl Acad Sci USA. 2014;111(43):15538-15543.
3. Anastasiadi D, et al. Consistent inverse correlation between DNA methylation of the first intron and gene expression across tissues and species. *Epigenetics Chromatin* 2018;11:37.
4. Jones PA. Functions of DNA methylation: islands, start sites, gene bodies and beyond. *Nat Rev Genet* 2012;13:484-492.
5. Barau J, et al. The DNA methyltransferase DNMT3C protects male germ cells from transposon activity. *Science*2016;354:909-912.
6. Lev Maor G, et al. The alternative role of DNA methylation in splicing regulation. *Trends Genet* 2015;31:274-280.

Reviewer #5 (Remarks to the Author):

I co-reviewed this manuscript with one of the reviewers who provided the listed reports. This is part of the Nature Communications initiative to facilitate training in peer review and to provide appropriate recognition for Early Career Researchers who co-review manuscripts.

We appreciate your time and effort.

REVIEWER COMMENTS

Reviewer #2 (Remarks to the Author):

For the most part the authors have done an excellent job of addressing our revisions. Thank you for carefully addressing our points and adding enrichment analyses, sensitivity analyses, multiple testing adjustment, supplemental data, and clarification where requested. Below we raise a few minor and easily addressable concerns, mostly related to 1) avoiding overstatement in the abstract/introduction, 2) comparisons to previous results, and 3) needed clarifications in the text of the manuscript.

We thank the reviewer for their careful re-evaluation of our manuscript and for their encouraging assessment that the remaining issues are minor and readily addressable. We have revised the manuscript and supplementary materials accordingly. Below, we respond point by point.

1) Avoid overstatement and unsupported statements in the abstract and the introduction
- The first sentence of the abstract is misleading in that it seems to suggest DNA methylation changes as a prime candidate to mediate the relationship between diet and health, when really the evidence presented for this is quite limited. Perhaps this can be toned down or removed.

Following the reviewer's suggestion, we revised the opening of the Abstract to avoid implying that DNA methylation is a prime mediator of the diet–health relationship. We removed the phrase "potentially through DNA methylation changes" from the first sentence, and reframed "whether they affect health outcomes through the same molecular mechanisms" to the more neutral "whether they are associated with similar DNA methylation profiles." The revised text now reads (page 3, lines 45–48): *" Several dietary patterns have been associated with better health outcomes. However, the extent to which adherence to different so-called 'healthy diets' overlaps, whether these dietary patterns are equally beneficial, and whether they are associated with similar DNA methylation profiles, remains unclear."*

- The final sentence of the abstract is improved but probably still stronger than warranted since a causal connection to health outcomes has not been established. How about the addition of the word "could" so that it reads "suggests that general adherence to a healthy dietary pattern could promote health"?

As suggested, we have added "could" to the concluding sentence of the Abstract to avoid implying a causal relationship beyond what is supported by the present data (page 3, lines 57–59). The revised sentence now reads: *"Our research thus suggests that general adherence to a healthy dietary pattern could promote health through similar epigenetic mechanisms, despite variations in dietary composition."*

- Refs 28 and 29 are described as showing evidence that "diet quality can significantly impact an individual's epigenome". However, the beta coefficients from associations reported in these papers are quite small, with only a few replicating across cohorts, suggesting subtle rather than significantly impactful changes. This wording could be toned down.

To better reflect the magnitude and nature of the reported associations in refs. 28(now 27) and 29 (now 28), we revised the sentence in the Introduction to avoid overstating the strength of the evidence (page 5,

lines 106–108): *"Indeed, recent work suggests that diet quality is associated with changes in DNA methylation across the epigenome,[27,28] which in part can be captured by epigenetic clocks that estimate biological age based on DNA methylation patterns."*

- It is unclear why ref 26 was cited; the topic and finding does not seem very related to the statement it is supposed to support. And neither reference 26 nor 27 supports use of the word "crucial" here.

We thank the reviewer for this observation. We have (i) softened the language by removing "crucial," (ii) removed reference 26, which we agree was not directly supportive of the statement, and (iii) replaced "interacts" with "may interact" to avoid overclaiming. The revised text now reads (page 4, lines 99–100): *"Epigenetic modifications to DNA represent a molecular mechanism through which environmental exposures, including diet, may interact with the genome."*

- The rest of the introduction seems to state findings accurately without overstatement.

2) Comparison to previous results
- Would be useful to indicate what "SeqId" refers to in header of Supplemental Table 8

We have clarified the meaning of "SeqId" in Supplementary Tables 8 and 18 by (i) updating the column header to indicate that SeqId refers to the protein assay identifier used in the SomaScan platform in the original study, and (ii) adding a corresponding footnote with the full definition: *"SeqId refers to the protein assay identifier used in SomaScan platform, as reported in the original study in the EWAS Catalog".*

- Would be useful to mention in Results that you're using the EWAS catalog to identify previous results; this will save the reader for having to hunt this down in Methods.

As suggested, we have revised the relevant paragraph in the Results to make explicit that we used the EWAS Catalog to look up previously reported associations (page 9, lines 269–271). The revised text now reads: " *A lookup in the EWAS Catalog revealed that several of the CpGs associated with MDS, DASH, MIND, AHEI, hPDI, and DII in our data had previously been reported in association with AHEI in other cohorts (Suppl. Table 18)."*

- Although some of this information may be in SuppTab 8 and mixed in with the other results, it would be very useful to separately examine (and describe in the text) whether any of your replicated EWAS CpGs overlap with CpGs reported in refs 28 or 29.

We thank the reviewer for this helpful suggestion. We have now explicitly examined whether any of our epigenome-wide significant EWAS CpGs overlap with CpGs reported by Ma et al.[1] (now ref 27) and Do et al.[2] (now ref 28), which used the Illumina 450K array.
Three CpGs identified in the Rhineland Study discovery cohort overlapped with the 24 AHEI-associated CpGs reported by Do et al.[2] in the Women's Health Initiative and TwinsUK cohorts: cg01676795 (*POR*), cg01004980 (*PRKAR2A*), and cg20954977 (*B3GNT7*). These three CpGs are present on both EPICv1 and 450K array. Of these, cg01676795 was successfully replicated in EPIC-Potsdam, showing a consistent effect direction and $p < 0.05$ for DASH, DII, hPDI, and MIND. cg01004980 and cg20954977 were partially replicated, with consistent effect directions but $p > 0.05$. For cg01676795 and cg01004980, the overlap included the same diet score (AHEI), whereas cg20954977 was associated with the MIND diet rather than AHEI in our cohort.

No overlap was observed between our epigenome-wide significant CpGs and the 10 MDS- or 24 AHEI-associated CpGs reported by Ma et al.[1] This lack of overlap is consistent with Do et al.'s own observation that their 24 replicated CpGs also did not overlap with those reported by Ma et al.. It may reflect methodological differences between studies: Ma et al. and Do et al. used the Illumina 450K array, whereas our study used the MethylationEPIC 850K v1 array in both cohorts. Some of our top hits are only on EPIC 850K v1 array. For instance, of the 13 epigenome-wide significant CpGs we identified for MDS only 4 are present on the 450K array, and of the 54 epigenome-wide significant CpGs identified for AHEI only 25 are on the 450K array.

We have added a brief summary of this lookup to the Results section (page 9, lines 271–289):
"Specifically, we examined overlap with the two prior EWAS of diet quality that applied similar hypothesis-driven scoring to population-based cohorts.[27,28] Three CpGs in our discovery cohort overlapped with the 24 AHEI-associated CpGs reported by Do et al.[28]: cg01676795 (POR), which was replicated in EPIC-Potsdam (consistent effect direction and $p < 0.05$) for DASH, DII, hPDI, and MIND; cg01004980 (PRKAR2A), which was partially replicated (consistent effect direction, $p > 0.05$) for AHEI, MDS, and MIND; and cg20954977 (B3GNT7), which was partially replicated for MIND. For cg01676795 and cg01004980, the overlap included the same diet score (AHEI), whereas cg20954977 was associated with MIND rather than AHEI in our cohort. No overlap was observed with the 10 MDS- or 24 AHEI-associated CpGs reported by Ma et al.[27]. This may reflect differences in methylation array coverage, as our study used MethylationEPIC v1 array, whereas the two previous studies used the 450K array."

References:
1. Ma, J. *et al.* Whole Blood DNA Methylation Signatures of Diet Are Associated With Cardiovascular Disease Risk Factors and All-Cause Mortality. *Circulation: Genomic and Precision Medicine* **13**, e002766 (2020).
2. Do, W. L. *et al.* Epigenome-wide association study of diet quality in the Women's Health Initiative and TwinsUK cohort. *Int J Epidemiol* **50**, 675–684 (2020).

- Supplemental Table 8 is referenced twice in the text; for the second reference I believe you intended to reference Supplemental Table 21.

On re-checking, Supplementary Tables 8 and 21 (now Suppl. Table 20) refer to two distinct but related analyses, and both citations in the paragraph are intentional. Supplementary Table 8, column "mQTL" lists the specific mQTLs identified for diet-associated CpGs through lookup in mQTLdb, whereas Supplementary Table 20 presents the enrichment analyses assessing whether EWAS-identified CpGs were over-represented among CpGs with known mQTLs, following your earlier suggestions. We hope this clarifies why both tables are cited in the relevant paragraph (page 10, lines 308–324).
However, during our review we identified and corrected mismatched references that had been introduced during the previous round of revisions:
- Page 6, line 158; Page 7, lines 174, 177, 181, 185: Suppl. Table **3**→ Suppl. Table **2**
- Page 7, line 175: Suppl. Table **2**→ Suppl. Table **3**
- Page 8, line 220**:** Suppl. Table **12** → Suppl. Table **11**
- Page 8, line 227**:** Suppl. Table **11** → Suppl. Table **12**
- Page 8, lines 228–230: Suppl. Table **6** → Suppl. Table **13**
- Page 9, lines 260: Suppl. Table **10** → Suppl. Table **17**
- Page 9, lines 266: Suppl. Table **18** → Suppl. Table **17**
- Pages 10, lines 301: Suppl. Table **4** → Suppl. Table **8**
- Page 10, lines 319: Suppl. Table **21** → Suppl. Table **20**
- Page 13, lines 361: Suppl. Table **20** → Suppl. Table **21**

We are grateful to the reviewer for prompting this thorough check.

3) Points satisfactorily addressed in response but requiring further clarification in paper. Here we agreed with the response but wanted to see further clarification of these points in the text. Here we have reprinted the original responses, with our recommendation for each point listed after "REVIEWER RESPONSE".

We appreciate the reviewer's suggestion to carry these clarifications into the manuscript and supplementary materials.

Line 368/Supplemental Figure 1– Why were participants of non-Caucasian descent removed (702)? Why did about 1000 have missing or doubtful FFQ data? (Supplemental Figure 1)?

We restricted analyses to participants of European ancestry to minimize confounding by ancestry-related differences in DNA methylation patterns and to ensure comparability with epigenetic clocks and EWAS reference resources, which have been predominantly developed and calibrated in European-ancestry populations. In addition, both the Rhineland Study and the EPIC-Potsdam participants are predominantly German of Caucasian descent. Given the relatively small number of non-European ancestry participants with complete data, we were not able to perform robust ancestry-stratified analyses.

The missing dietary assessment (n=841) cases were participants who did not complete the FFQ at baseline (i.e., no dietary questionnaire data were available). Following the transition to the web-based format, the FFQ was completed predominantly at home via an emailed link. Participants who reported being unable to complete it at home were offered an additional appointment at the study center. Nevertheless, because completion occurred outside the study center for most participants, we cannot infer specific reasons for non-completion.

Among participants with baseline dietary assessment, incomplete or doubtful FFQ data (n=200) reflects questionnaires that did not meet our predefined quality criteria. Specifically, n=197 did not complete at least 80% of FFQ items (the completion threshold we have adopted, similarly to previous studies)[1], and n=3 were excluded during quality assurance due to concerns about questionnaire validity (e.g., insufficient German language proficiency flagged by study staff). We updated the flowchart of included participants to reflect this distinction (Supplementary Figure S1).

Reference:

1. Knüppel, S., Clemens, M., Conrad, J. et al. Design and characterization of dietary assessment in the German National Cohort. Eur J Clin Nutr 73, 1480–1491 (2019). https://doi.org/10.1038/s41430-018-0383-8

REVIEWER RESPONSE: Satisfactory explanation. At a minimum, please include footnotes to Supplementary Figure 1 addressing these points. If there is space, please include at least one sentence addressing each point in the manuscript methods.

We have addressed this in two complementary places:

(*i*) Supplementary Figure 1 footnote. We expanded the footnote to clarify each exclusion category. Specifically, we now explain (a) the rationale for restricting analyses to participants of European ancestry, including why ancestry-stratified analyses were not feasible; (b) that "missing dietary assessment" refers to participants who did not complete the FFQ at baseline, and that specific reasons for non-completion could not be systematically recorded because most participants completed the questionnaire remotely via a web-based format. In addition, to characterize potential differences between FFQ non-responders and our analytical population, we have now added a dedicated column to Supplementary Table S1 comparing baseline characteristics of these two groups. Compared with the analytical population, FFQ non-responders differed only modestly in demographic characteristics. They were somewhat more likely to be male (48% vs 43%) and slightly younger on average (mean age 55.3 vs 56.3 years), with a small overrepresentation in the 30–49 age groups. FFQ non-responders also had marginally lower educational attainment, slightly higher BMI (mean 26.4 vs 25.9 kg/m²), and a marginally higher prevalence of diabetes. Patterns broadly consistent with those observed for the overall non-included population; (c) that "incomplete FFQ data" refers to questionnaires with fewer than 80% of FFQ items completed, and "QA excluded" refers to questionnaires excluded during quality assurance (e.g., insufficient German language proficiency); and (d) that "implausible caloric intake" refers to participants with reported total energy intake outside a predefined plausibility range.

The full revised footnote reads: "*"Missing dietary assessment" refers to participants who did not complete the FFQ at baseline; Most participants completed the questionnaire at home in a web-based format, and therefore specific reasons for non-completion cannot be inferred. "Incomplete FFQ data" refers to questionnaires with fewer than 80% of FFQ items completed; "QA excluded" refers to questionnaires excluded during quality assurance (e.g., due to concerns about questionnaire validity such as insufficient German language proficiency). "Implausible caloric intake" refers to participants with reported total energy intake outside a predefined plausibility range (< 800 or > 6000 kcal/day). Participants without available genetic data or classified as being of non-European ancestry based on genetic principal components were further excluded from the EWAS. This ancestry restriction was applied to reduce potential confounding by ancestry-specific differences in DNA methylation patterns."*

(ii) Methods section. We added a brief clarification in the Study Population paragraph (page 17, lines 490–495), directing readers to Supplementary Figure 1 for the full exclusion flow and stating the rationale for the ancestry restriction. The revised passage now reads*: "Of these, 5768 participants with available genetic data and of European ancestry were further included in our EWAS; the ancestry restriction was applied to reduce confounding by ancestry-specific differences in DNA methylation patterns. Details on the stepwise exclusion of participants with missing or incomplete dietary assessment, epigenetic data, or covariate information are provided in Supplementary Figure 1."*

We also specifically mention that most of the participants answered the FFQ in a web-based format (page 17, lines 483–485): "*The baseline dietary assessment is completed remotely in a web-based format by most participants, within a defined window following the study centre visit, during which blood samples for DNA methylation analysis are drawn."*

Line 376 – Does this mean that participants needed to have responses for at least 80% of the food items in the FFQ food list?

Yes. For inclusion in the analyses, participants were required to provide responses for at least 80% of FFQ food and beverage items. FFQs below this completeness threshold were considered incomplete and excluded[1] (corresponding to the n=197 exclusions shown in Supplementary Figure 1).

Reference:
1. Knüppel, S., Clemens, M., Conrad, J. et al. Design and characterization of dietary assessment in the German National Cohort. Eur J Clin Nutr 73, 1480–1491 (2019). https://doi.org/10.1038/s41430-018-0383-8

REVIEWER RESPONSE: Satisfactory explanation. Please also include a sentence or two specifying this in the manuscript.

We thank the reviewer for this suggestion. The 80% completeness criterion was already specified in the Methods; to make this more explicit, we have added a follow-up sentence clarifying that questionnaires below this threshold were excluded (page 17, lines 499–502). The passage now reads: *"For analysis inclusion, FFQs needed data on at least 80% of food items, consistent with other cohort studies using a similar instrument.[47] Questionnaires not meeting this completeness threshold were considered incomplete and excluded."* Reference 47 corresponds to the Knüppel et al. (2019) study cited in our previous response.

Line 376 – Were the FFQ measures collected during the same time point as the buffy coat samples for both Rhineland and EPIC-Potsdam?

In both the Rhineland Study and EPIC-Potsdam, the FFQ and buffy coat samples were collected at the same time during the baseline assessment.

REVIEWER RESPONSE: Satisfactory explanation. Please also specify in the manuscript.

We have now specified the timing of FFQ administration relative to buffy coat sample collection in both cohorts.
For the Rhineland Study, we added this information to the Study Population section (page 17, lines 481–483). The relevant passage now reads: *"The baseline dietary assessment is completed remotely in a web-based format by most participants, within a defined window following the study centre visit, during which buffy coat samples for DNA methylation analysis are drawn."*
For EPIC-Potsdam, we added this information to the Validation in an independent cohort section (page 21, lines 626–627). The relevant sentence now reads: *"In the EPIC-Potsdam, both dietary assessment and buffy coat collection were performed at baseline."*

Line 168 – Why only 1k people from EPIC Potsdam? Was this not a much larger study? Who was included?

The EPIC-Potsdam study is indeed a large population-based cohort with approximately 27,000 participants.[1] DNA methylation was measured using the Illumina MethylationEPIC v1.0 array in a randomly selected subcohort of EPIC-Potsdam (n= 1147) as part of a nested case-cohort design. [2] The 1,034 participants included in our replication analysis represent those with available EPIC v1.0 array methylation data and complete dietary and covariate information at the time of our analysis. This subsample is broadly representative of the overall EPIC-Potsdam cohort in terms of key demographic characteristics. The sample size is consistent with other recent methylation studies from EPIC-Potsdam[2] and provides adequate statistical power for replication of epigenome-wide findings, as demonstrated by our successful validation of associations for diet quality scores with epigenetic aging markers and CpG sites.

References:
1. Boeing H, et al. Recruitment procedures of EPIC-Germany. Ann Nutr Metab. 1999;43(4):205-15.
2. Eroglu, B., Eichelmann, F., Kuxhaus, O. et al. Trace element-linked DNA methylation sites and their association with type 2 diabetes and cardiovascular diseases: EPIC-Potsdam cohort study. Clin Epigenet 17, 172 (2025).

REVIEWER RESPONSE: Were the sub-sample characteristics comparable to the overall study population? Selection bias among those who had methylation data?

The n = 1,034 analytical sample is a randomly selected subcohort of EPIC-Potsdam (n = 1,147), which was assembled as part of a nested case-cohort design.[1] A core statistical property of the case-cohort design is that the randomly sampled subcohort is representative of the parent cohort by construction.[2] This design feature directly addresses concerns about selection bias in methylation-based analyses.

The table below compares the main demographic baseline characteristics between the full EPIC-Potsdam cohort and n = 1,034 analytical sample used for replication in our study:

		EPIC-Potsdam cohort (n=27,301)	Replication sub-sample (n=1,034)
Age, mean y ± SD		50.5 ± 9.0	49.88 ± 8.9
Women, n (%)		16,501 (60)	640 (61.9)
Education ISCED11, n (%)	High	9,971 (37)	402 (38.9)
	Medium	6,772 (25)	247 (23.9)
	Low	10,558 (39)	385 (37.2)
BMI, mean kg/m^2 ± SD		26.3 ± 4.4	25.82 ± 4.0
Smoking status, n (%)	Never	12,842 (47)	509 (49.2)
	Former	8,834 (32)	315 (30.5)
	Current	5,625 (21)	210 (20.3)

This subsample is broadly representative of the overall EPIC-Potsdam cohort in terms of key demographic characteristics. We have additionally clarified in the manuscript that the replication sample derives from a randomly selected subcohort of EPIC-Potsdam (page 21, lines 616–621) *"The replication sample was a randomly selected subcohort of EPIC-Potsdam (n = 1,147) assembled as part of a nested case-cohort design; the 1,034 participants included in our replication analysis were those with available methylation data and complete dietary and covariate information. This subsample is broadly representative of the overall EPIC-Potsdam cohort in terms of key demographic characteristics (**Suppl. Table 9**)"*, alongside the existing demographic characteristics reported in Supplementary Table 9.

References:
3. Eroglu, B., Eichelmann, F., Kuxhaus, O. et al. Trace element-linked DNA methylation sites and their association with type 2 diabetes and cardiovascular diseases: EPIC-Potsdam cohort study. Clin Epigenet 17, 172 (2025).
4. Prentice RL. A case-cohort design for epidemiologic cohort studies and disease prevention trials. Biometrika. 1986;73(1):1–11.

Reviewer #3 (Remarks to the Author):

I co-reviewed this manuscript with one of the reviewers who provided the listed reports. This is part of the Nature Communications initiative to facilitate training in peer review

and to provide appropriate recognition for Early Career Researchers who co-review manuscripts.

Thank you very much for your time and effort.

Reviewer #5 (Remarks to the Author):

I co-reviewed this manuscript with one of the reviewers who provided the listed reports. This is part of the Nature Communications initiative to facilitate training in peer review and to provide appropriate recognition for Early Career Researchers who co-review manuscripts.

We appreciate your time and effort.
